# Assessment of the Geographic Distribution of *Ornithodoros turicata* (Argasidae): Climate Variation and Host Diversity

**DOI:** 10.1371/journal.pntd.0004383

**Published:** 2016-02-01

**Authors:** Taylor G. Donaldson, Adalberto A. Pèrez de León, Andrew I. Li, Ivan Castro-Arellano, Edward Wozniak, William K. Boyle, Reid Hargrove, Hannah K. Wilder, Hee J. Kim, Pete D. Teel, Job E. Lopez

**Affiliations:** 1 Department of Entomology, Texas A&M AgriLife Research, College Station, Texas, United States of America; 2 United States Department of Agriculture–Agricultural Research Service, Knipling-Bushland U.S. Livestock Insects Research Laboratory and Veterinary Pest Genomics Center, Kerrville, Texas, United States of America; 3 United States Department of Agriculture–Agricultural Research Service, Invasive Insects Biocontrol and Behavior Laboratory, Beltsville, Maryland; 4 Department of Biology, Texas State University, San Marcos, Texas, United States of America; 5 Texas State Guard, Medical Brigade, Uvalde, Texas, United States of America; 6 Department of Biological Sciences, Mississippi State University, Starkville, Mississippi, United States of America; 7 Department of Pediatrics, National School of Tropical Medicine, Baylor College of Medicine, Houston, Texas, United States of America; University of California, San Diego School of Medicine, UNITED STATES

## Abstract

**Background:**

*Ornithodoros turicata* is a veterinary and medically important argasid tick that is recognized as a vector of the relapsing fever spirochete *Borrelia turicatae* and African swine fever virus. Historic collections of *O*. *turicata* have been recorded from Latin America to the southern United States. However, the geographic distribution of this vector is poorly understood in relation to environmental variables, their hosts, and consequently the pathogens they transmit.

**Methodology:**

Localities of *O*. *turicata* were generated by performing literature searches, evaluating records from the United States National Tick Collection and the Symbiota Collections of Arthropods Network, and by conducting field studies. Maximum entropy species distribution modeling (Maxent) was used to predict the current distribution of *O*. *turicata*. Vertebrate host diversity and GIS analyses of their distributions were used to ascertain the area of shared occupancy of both the hosts and vector.

**Conclusions and Significance:**

Our results predicted previously unrecognized regions of the United States with habitat that may maintain *O*. *turicata* and could guide future surveillance efforts for a tick capable of transmitting high–consequence pathogens to human and animal populations.

## Author Summary

Argasid ticks are understudied vectors of significant human and veterinary pathogens. The life-cycle and feeding behavior of the tick poses challenges when attempting to understand the vector’s distribution. These ticks reside in dens, nests, and cave cavities, and are indiscriminant nocturnal feeders. They also engorge within minutes of attachment, and identifying the ticks on a vertebrate host is infrequent. To guide future surveillance studies, we predicted regions of probable occurrences for *Ornithodoros turicata*, a species capable of transmitting relapsing fever spirochetes and African swine fever virus. Historical databases and published literature were evaluated, and we collected ticks from regions of the United States. Environmental factors linked with known localities of *O*. *turicata* were used in a mathematical modeling program, which predicted regions in the United States and north Mexico likely to sustain the ticks. Additionally, vertebrate host ranges were associated with the predictive models, which may indicate how the tick vectors are dispersed. Collectively, these studies identified previously unrecognized regions that could sustain the ticks, and we envision that our work will help guide surveillance and additional research efforts to understand the ecology of pathogens transmitted by argasid ticks.

## Introduction

Argasid ticks in the genus *Ornithodoros* are globally distributed with five known species present in the United States. In the western and midwestern regions of the United States, *Ornithodoros coriaceus*, *Ornithodoros hermsi* and *Ornithodoros parkeri* are distributed in various ecological settings, while *Ornithodoros turicata* and *Ornithodoros talaje* are found in arid regions of the southern United States and into Latin America [1,2]. *Ornithodoros* ticks are known vectors of veterinary and medically significant pathogens [3]. The ticks transmit spirochetes that cause relapsing fever borreliosis [4,5], while *O*. *coriaceus*, *O*. *parkeri*, and *O*. *turicata* have also been experimentally infected with African swine fever virus (ASFV) [6]. With outbreaks occurring in Central and South America, and the Caribbean, ASFV is considered a global threat to the livestock industry [7]. The distribution of *Ornithodoros* species, particularly *O*. *turicata* in the southern United States, indicates that a considerable opportunity exists for the maintenance of ASFV were the pathogen introduced [8].

*Ornithodoros turicata* was first described from specimens collected in Guanajuato, Mexico in 1876 [4,9], while collections in the United States include Arizona, California, Colorado, Florida, Kansas, New Mexico, Oklahoma, Texas, and Utah [4,8,10]. The absence of *O*. *turicata* collections between Texas and Florida indicates the Florida population may be geographically isolated [4,8,10–14]. Moreover, the distribution of the *O*. *turicata* throughout regions of North America suggests the potential for considerable adaptability and genetic plasticity.

The feeding behavior of *O*. *turicata* further indicates the adaptability of the ticks. *Ornithodoros turicata* are nidicolous nocturnal feeders [4,14] that engorge within 60 minutes, and are rarely found attached on their vertebrate hosts [15,16]. They colonize peridomestic settings and inhabit burrows, nests, caves, and cavities under outcroppings [4,15]. *Ornithodoros turicata* are also promiscuous feeders and recognized hosts include prairie dogs (*Cynomys* spp.), ground squirrels (*Spermophilus* spp.), snakes, cattle, pigs, and the gopher tortoise (*Gopherus polyphemus*) [4,15]. Moreover, successful laboratory colonies have been produced by feeding the ticks on mice [17] and chickens (authors JEL and PDT), while reports from Texas associate the arthropods with feeding on canines and humans [18,19].

The life cycle of *O*. *turicata* contributes to the capability of the tick to serve as a reservoir for pathogens. The arthropods can have up to six nymphal instars and survive extreme periods between bloodmeals [17]. In laboratory studies, the life span of adult ticks was over 10 years when fed regularly, and they endured at least five years between feedings [4,20,21]. Prolonged periods of starvation in nature were also reported in mark-and-recapture studies [12]. Adults also feed and reproduce multiple times throughout their life time. The longevity and hardiness of *O*. *turicata* indicates the importance of understanding ecological factors that may impact the distribution of the ticks.

Linkages of biotic and abiotic factors to tick distribution, and risk of exposure to tick-borne pathogens have been most investigated and modeled for surface dwelling ixodid ticks [22,23]. Nidicolous argasid ticks present an additional challenge for modeling, as their life history plays out in niches between subterranean microclimates of burrows or landscape cavities (e.g., caves) and the external environment [24]. Tick development, survival, and dispersal are interdependent upon the population dynamics, diversity, resting cycle, and surface activity of their vertebrate hosts [25]. This current study aimed to provide a composite prediction of the distribution of *O*. *turicata* in the United States and into north Mexico. We generated locality points for *O*. *turicata* at the county-level by evaluating historic databases and performing tick collection efforts. The data were used in conjunction with a maximum entropy species distribution model (Maxent), which offers good performance, robustness, and statistical validation [26–28]. A shapefile depicting the predicted distribution of *O*. *turicata* was generated based on the most informative Maxent model. This shapefile was overlaid with the range of the community of hosts in order to obtain the area shared between individual vertebrate species and *O*. *turicata*. Our report provides the framework that will guide future tick surveillance efforts and research to further understand host communities and suitable habitat that supports the maintenance of *O*. *turicata*.

## Methods

### Ethics statement

Tick baiting collections from *G*. *polyphemus* dens in Florida were performed under permit number 2014–0310 from the Florida Department of Agriculture and Consumer Services. When *O*. *turicata* specimens were collected from Wildlife Management Areas in Texas (WMA), approval was obtained from Texas Parks and Wildlife and WMA personnel.

### Database search and field collection of *O*. *turicata*

The currently known distribution of *O*. *turicata* was assessed by performing literature searches using *O*. *turicata* and *B*. *turicatae* as key words (*n* = 47) [13,19,20,29–35]. When authors reported specific regions of field collected ticks or locations of human exposure to *B*. *turicatae* geographic data points were noted at the county level. Records of *O*. *turicata* collections were also obtained from the United States National Tick Collection (USNTC) [36] and the Symbiota Collections of Arthropods Network (SCAN) (*n* = 104) [37], and field studies in Texas and Florida (*n* = 7). The catalog of *O*. *turicata* collections provided from the USNTC consisted of *O*. *turicata* numbers, developmental stages, specimen identification, and locality information. When a municipality, county, or estimated location of collection was provided, manually georeferenced data points were generated using Google Earth [38]. When ticks were collected from a vertebrate host, the level of host taxa was noted.

When *Ornithodoros* ticks were collected at field sites in Texas and Florida, the latitude and longitude were recorded. In the evening, dry ice was placed at the openings of gopher tortoise burrows, coyote (*Canis latrans*) dens, and within 1–2 m of rodent (*Neotoma*, *Peromyscus*, and *Sigmodon* spp.) and burrowing owl (*Athene cunicularia*) nests. As ticks emerged from a given location, they were collected and housed in 15 or 50 ml conical tubes with perforated caps, and kept separate according to the coordinates of the den and nest. Upon returning to the laboratory, the arthropods were housed at 27°C and 85% and 95% relative humidity for ticks collected in Texas and Florida, respectively [39,40]. They were also inspected by microscopy to determine species collected using taxonomic descriptions [4].

### Species Distribution Models (SDMs)

A total of 158 *O*. *turicata* localities with no duplicates from the datasets described above were used to build the SDMs. Locality records (presence only data) that lacked geographical coordinates (longitude and latitude) were georeferenced using GEOLocate v.3.21 [41]. For specimens that only had county level data, GEOLocate was used to obtain geographic coordinates from the centroid of each corresponding county. Twenty data layers containing environmental and altitude variables at 30 arc-seconds (≈1 × 1 km^2^ grid cells) spatial resolution (S1 Table) were acquired from the WorldClim dataset (http://worldclim.org) [42]. The WorldClim dataset was generated through interpolation of average monthly data from numerous global weather stations representing a time period from 1950–2000. To determine whether there were correlations among the environmental variables, a correlation matrix was built using the “Explore Climate: Correlations and Summary Stats tool” within an extension toolkit SDMtoolbox (http://sdmtoolbox.org) [43]. Based on the Pearson’s correlation value we eliminated one variable in each pair that was either ≥ 0.80 or ≤ -0.80 (S2 Table). The variable accepted out of the pair was based on biological importance in relevance to *O*. *turicata*. Out of the 20 variables we kept 12 (Table 1). Localities, environmental, and altitude variables were used with a common machine learning technique, maximum entropy modeling (Maxent v.3.3.3k) [26] to construct four *O*. *turicata* SDMs. Maxent was chosen because of its performance, robustness, and statistical validation [27,28]. Moreover, given the limited understanding of *O*. *turicata* geographic range and Maxent’s use of presence-only data, this modeling approach was an appropriate first step to increase our understanding of the distribution of the tick. Maxent was set up with the following default settings: algorithm parameters set as auto features, a convergence threshold of 0.00001, a maximum of 10,000 background points, a regularization multiplier of 1, and a logistic output grid format; all other remaining parameters were left in the default settings. In addition to these default settings the number of iterations was modified to 5,000. Response curves were also included for each Maxent model run, which allows one to evaluate how the prediction depends on the variables. Maxent creates two types of response curves, the first shows how the prediction changes with each variable while keeping all other variables in the model (S1 Fig), and the second only utilizes the corresponding variable (S2 Fig). The advantage of the second response curves is that it allows for easier interpretation of environmental variables that may be correlated or slightly correlated. The following four models with 10 subsampling runs using a random test percentage of 20% (*n* = 31) were constructed with predefined environmental and altitude parameter data layers (Table 1): 1) an average precipitation model, 2) an average temperature model, 3) an average full model which included all environmental variables and altitude, and 4) an average model based on the top-five environmental variables from the full model (top-five model).

**Table 1:** Climate and altitude variables for the four species distribution models of *O*. *turicata* constructed using Maxent.

Climate and Altitude Variables	Variable Code	Precipitation Model	Temperature Model	Full Model	Top-five Model
Annual Mean Temperature	BIO1	-	X	X	-
Mean Diurnal Range	BIO2	-	X	X	-
Temperature Seasonality	BIO4	-	X	X	-
Max Temperature of Warmest Month	BIO5	-	X	X	X
Mean Temperature of Wettest Quarter	BIO8	-	X	X	X
Mean Temperature of Driest Quarter	BIO9	-	X	X	X
Annual Precipitation	BIO12	X	-	X	-
Precipitation Seasonality	BIO15	X	-	X	X
Precipitation of Driest Quarter	BIO17	X	-	X	-
Precipitation of Warmest Quarter	BIO18	X	-	X	X
Precipitation of Coldest Quarter	BIO19	X	-	X	-
Altitude	ALT	-	-	X	-

Maxent calculated the area under the curve (AUC), a value that ranges from 0.5 to 1, providing information on the species’ restricted predicted distribution in relation to the range of predictor variables in the model, but this value does not necessarily measure the fit of the model [44]. An informative AUC score is equal to 1, while an AUC score of 0.5 equates a model performance no better than random. To see which variables contributed the most to the model, Maxent calculated the percent contribution (PC) and permutation importance (PI) of each variable for all models. The PC value is dependent on the algorithm path that Maxent used to obtain the model while PI depends only on the final Maxent model. Additionally, variable jackknifing for the training gain, test gain, and test AUC was also conducted where each environmental variable was excluded. A model was subsequently created, and the process was repeated until all the variables had been excluded. The second part of the jackknife test created a model using each environmental variable in isolation of one another. Specifically for the top-five model, three additional models were run using: 1) the top-five variables from the PC, 2) the top-five variables from the PI, and 3) the top-five variables from the jackknife results. The highest AUC score among these three models was selected to be our top-five environmental model.

All SDMs were processed and visualized in QGIS v.2.4 Chugiak [45] with the geographic area restricted to the United States and Mexico based on collection records available of *O*. *turicata*. Five classes of probabilities each with about a 20% interval were given a specific name and color for visual representation of model results: very high probability (red), high probability (orange), moderate probability (yellow), low probability (green), and very low probability (white).

A shapefile representing the most informative SDM was drawn in QGIS by including the percentage of low (< 20%) to very high probability (∼86%). To create a shapefile we excluded isolated areas that were not in close proximity from areas > ~20% (such as the states of Washington, Idaho, Montana, Wyoming, and South Dakota). Alabama, Louisiana, and Mississippi were also excluded because previous survey data indicated the absence of *O*. *turicata* [46]. This shapefile was then imported into ArcMap 10.2.2 [47] where the geometry of the shapefile was checked using the “Check Geometry Tool” within the “Data Management Toolbox.” This tool reported if any errors occurred, such as overlaps and/or no closed connection for polygons. If problems were found with the shapefile it was fixed using the “Repair Geometry Tool” also within the “Data Management Toolbox.” The shapefile was projected to the map projection of North American Albers Equal Area before the area (km^2^) of the shapefile was calculated.

### GIS analyses of host diversity and distribution

Host diversity was obtained from the USNTC, where host records were classified to either genus or species. Therefore in cases with taxa at the genus level we included all species known to occur in the most informative Maxent model distribution. In addition, we included 48 suspected hosts because these species are part of the burrow community utilizing the burrows of original excavators. For example *A*. *cunicularia* inhabits the burrows of *Cynomys ludovicianus* (S3 Table) [48]. A total of 58 host species were included in this study (S3 Table). Taxonomic names were based on the International Union for Conservation of Nature (IUCN) Red List of Threatened Species (http://iucnredlist.org) [49] and Wilson & Reeder’s Mammal Species of the World, 3^rd^ edition database [50]. We obtained most of the host species distribution shapefiles from the IUCN Red List of Threatened Species [49]. For *Sus scrofa*, its distribution was obtained from the National Feral Swine Mapping System, Southeastern Cooperative Wildlife Disease Study. No spatial data were provided in the IUCN Red List of Threatened Species for *G*. *agassizii* and *G*. *polyphemus* thus they were drawn in QGIS following known species distributions [51,52].

In ArcMap all shapefiles were checked and fixed for errors in geometry and projected to North American Albers Equal Area Conic. Some of the hosts’ distribution shapefiles obtained were clipped to the extent of the United States and Mexico. The area of extent (km^2^) for each host species’ distribution was calculated in ArcMap. Each of the host species shapefiles were intersected with the most informative SDM shapefile in order to obtain new shapefiles showing shared occupancy. For *A*. *cunicularia*, because of its nesting phenology, analyses were conducted with its full range and three range subdivisions: year-round, breeding, and winter. In ArcMap we calculated the area (km^2^) of these shared occupancies for each individual species. Next we calculated the percentage of the area shared for each species as $\%\;of\;area\;shared\;=\;\left(\frac{Area\;of\;individual\;spp.\;shared\;distribution}{Total\;area\;of\;O.\;turicata\;distribution}\;\times \;100\right)$.

## Results

### Defining *O*. *turicata* localities

**Fig 1:**
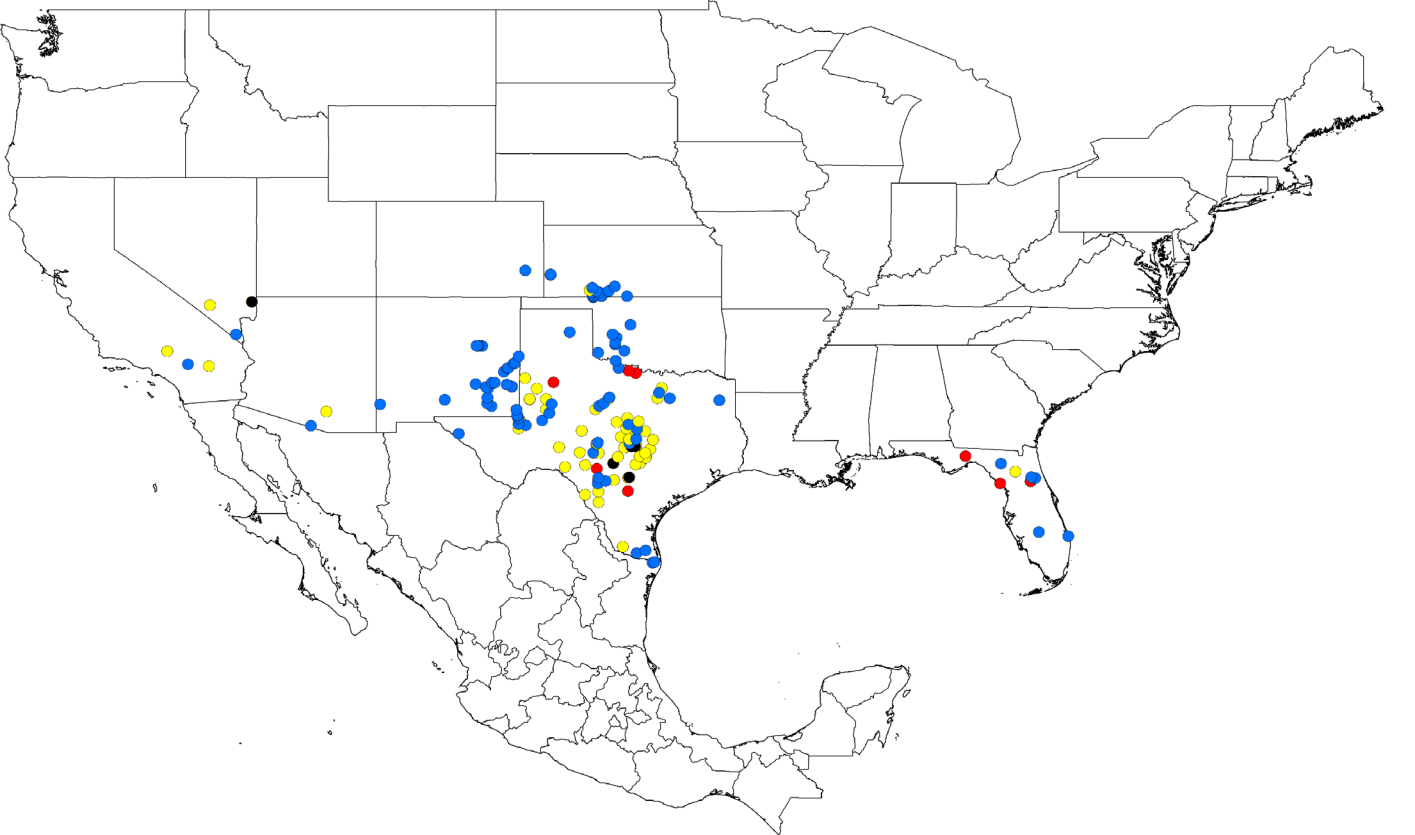
*Ornithodoros turicata* localities generated from USNTC and SCAN reports, literature reviews, and field studies. Localities in which a collection date was not recorded are represented by black circles. Blue, yellow, and red circles represent localities that were collected from 1800–1950, 1950–2000, and 2000-present, respectively.

A total of 158 sample localities were obtained by performing literature searches in the United States National Library of Medicine (*n* = 47) (S4 Table) [18–20,29,30,32,33,53], evaluating records provided by the USNTC and SCAN (*n* = 104), and field collection studies (*n* = 7) (Fig 1). Since tick-borne relapsing fever spirochetes are transmitted by a specific species of *Ornithodoros* [54], literature searches identified presumed case reports of infection caused by *B*. *turicatae* as determined by visualizing spirochetes in human blood smears, and county localities were included if *O*. *turicata* was collected [18,19,26]. Also, studies reporting molecular evidence of host infection with *B*. *turicatae* were used to define the distribution of the tick vector. For example, DNA sequence analysis demonstrated *B*. *turicatae* as the etiological agent in domestic dogs, and serological evidence indicated exposure of a human patient in central Texas [32,55]. These reports provided additional localities for *O*. *turicata*. Moreover, recent field studies from 2012–2014 in Texas and Florida resulted in the collection of *O*. *turicata* (Table 2), and indicated recent evidence of geographic area for this tick species.

**Table 2:** Collection summary of *O*. *turicata* in Texas and Florida.

Source	No. and stage^A^	Year	Locality^B^
Rodent nest	40L	2012 and 2013	Dimmit Co., Texas
Rodent nest	4N	2014	Frio Co., Texas
Coyote dens	8N	2013	Gonzales Co., Texas
Coyote dens and rodent nest	> 100A, >300N	2013–2014	La Salle Co., Texas
Rodent nest	2A	2014	Uvalde Co., Texas
Gopher tortoise den	20A	2014	Leon Co., Florida
Gopher tortoise den	20N, 10A	2013 and 2014	Ocala Co., Florida

^A^ L, larvae; N, nymph; A, adult

^B^ Co., county

### Maxent analysis

**Fig 2:**
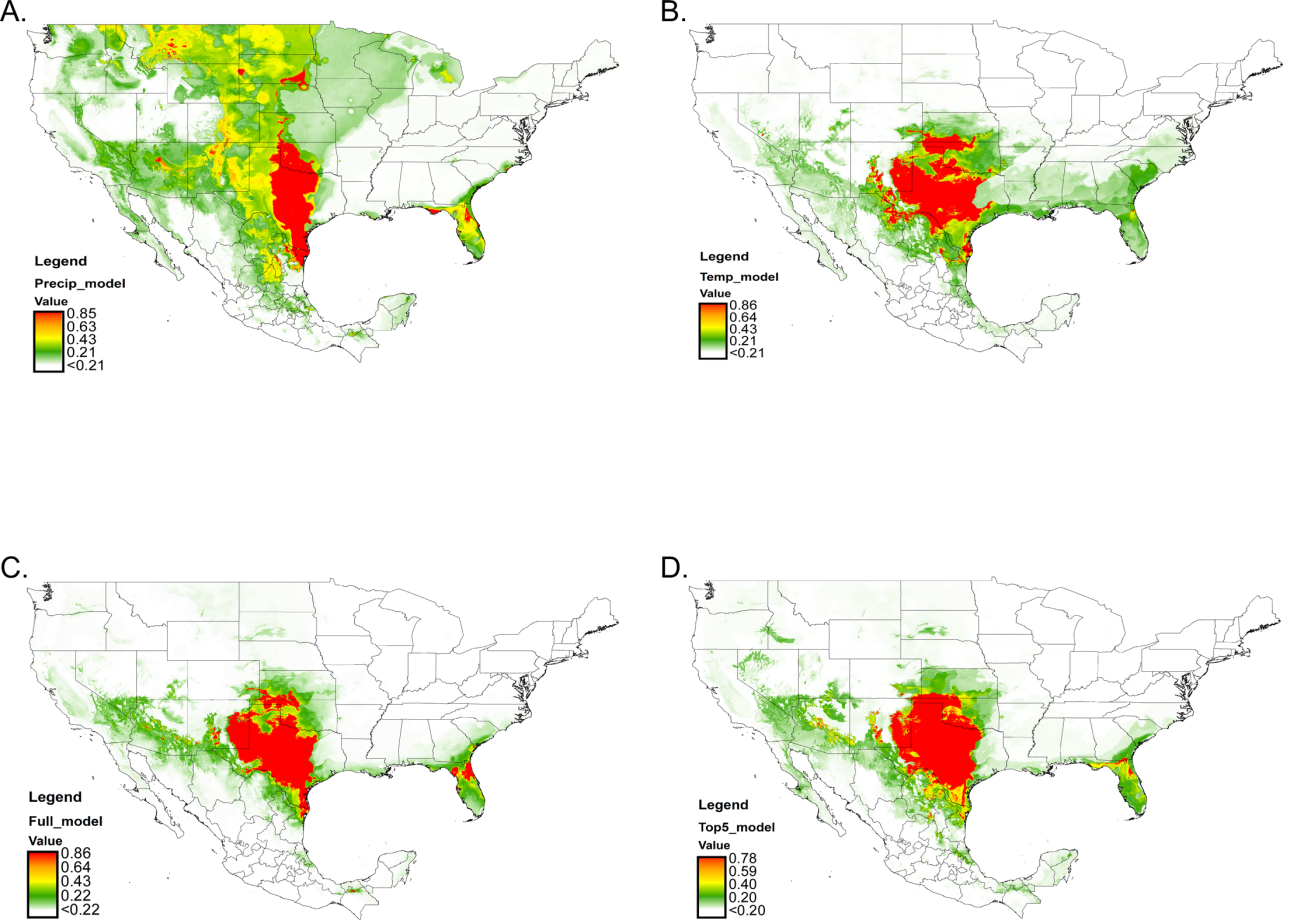
Model predictions for the distribution of *O*. *turicata* based on current climate conditions: A) precipitation model, B) temperature model, C) full model, and D) top-five environmental variables model. Probability of *O*. *turicata* classified into 5 categories: very high probability (red), high probability (orange), moderate probability (yellow), low probability (green), and very low probability (white).

The four average species distribution models produced by Maxent (precipitation, temperature, full, and top-five) show variation in the probability of occurrences for *O*. *turicata* within the United States and Mexico (Fig 2A–2D). The average AUC scores for the full, temperature, and precipitation models were 0.949 ± 0.011 SD, 0.925 ± 0.013 SD, and 0.910 ± 0.027 SD, respectively. The average AUC scores for the three top-five models are as follows: top-five environmental variables based on the percent contribution (PC) = 0.942 ± 0.013 SD, the permutation importance (PI) = 0.939 ± 0.010 SD, and the jackknifes 0.933 ± 0.01 3 SD. We chose the top-five model using the PC as is it had the highest AUC score out of the three. Thus the results to follow of our top-five model will focus on the top-five variables based on the PC. The regularized training gain is similar to a goodness of fit test, and at the start of a run for a given model this value begins at 0 and increases to an asymptote starting with a uniform distribution then gradually increasing the fit. When the model has reached its end the last gain value represents how the model fits around the input presence data. The highest average training gain was 2.065 ± 0.041 SD for the full model and with the average likelihood of the presence data 7.885 times higher than that of a random background pixel (*e*^2.065^ = 7.885). This average training gain value for the full model was followed by the value for the top-five (gain = 1.927 ± 0.050; times higher than random = 6.869), temperature (1.873 ± 0.032; 6.508), and precipitation (1.266 ± 0.061; 3.547). The average test gains followed the same trend with the full model having the highest test gain (2.021 ± 0.012 SD) followed by top-five (1.918 ± 0.252 SD), temperature (1.666 ± 0.187 SD), and precipitation (1.448 ± 0.266 SD).

In the average precipitation model, precipitation of warmest quarter (BIO18) was a major determining factor for percent contribution (PC) followed by precipitation seasonality (BIO15), annual precipitation (BIO12), precipitation of driest quarter (BIO17), and precipitation of coldest quarter (BIO19) (Table 3). As for permutation importance (PI), precipitation seasonality (BIO15), precipitation of the warmest quarter (BIO18), and precipitation of the driest quarter (BIO17), were most important for the full model (Table 3). The jackknife test of variable importance for the training gain, test gain, and test AUC indicated that the environmental variable with the highest gain when used alone was precipitation of the warmest quarter (BIO18) (S3A Fig). However, the environmental variable precipitation seasonality (BIO15) indicated the greatest decrease in gain when it was omitted, thus appearing to have the most information that is not present in other environmental variables. The variable in the precipitation model that produced the lowest training gain when used alone was precipitation of the coldest quarter (BIO19).

**Table 3:** Summary of environmental variables, of the average percent contribution (PC) and permutation importance (PI) for each of the four species distribution models of *O*. *turicata*.

Variables	Precipitation Model		Temperature Model		Full Model		Top-five Model	
	PC	PI	PC	PI	PC	PI	PC	PI
BIO1	-	-	5.24	33.75	6.14	29.07	-	-
BIO2	-	-	1.39	3.46	1.50	1.83	-	-
BIO4	-	-	7.74	13.32	4.24	9.59	-	-
BIO5	-	-	46.89	17.95	32.42	1.97	39.57	59.16
BIO8	-	-	15.24	3.06	15.86	0.87	16.96	2.82
BIO9	-	-	23.49	28.47	13.09	5.43	17.97	13.97
BIO12	7.66	8.39	-	-	2.89	30.41	-	-
BIO15	39.96	40.42	-	-	12.33	4.50	13.12	15.55
BIO17	4.16	19.42	-	-	0.59	9.6	-	-
BIO18	44.64	26.62	-	-	10.56	2.18	12.37	8.51
BIO19	3.58	5.15	-	-	0.15	2.31	-	-
ALT	-	-	-	-	0.22	2.24	-	-

The raster map of the precipitation model covered most of the Great Plains predicting a larger portion of probable regions in the central northern states (Montana, Nebraska, North Dakota, and South Dakota) (Fig 2A), an area where no confirmed records exist. In addition, the precipitation model predicted that many of our confirmed western (i.e., California, Nevada) and Florida localities were of low probability (~20%) for *O*. *turicata*. Thus, precipitation variables alone do not accurately reflect the distribution of field collected *O*. *turicata*, but suggests a geographical gap exists between Florida and Texas populations, and predicts a large area for which currently no records of *O*. *turicata* exist.

In the average temperature model, PC was highest for maximum temperature of warmest month (BIO5), followed by mean temperature of driest quarter (BIO9), annual mean temperature (BIO1), mean temperature of the wettest quarter (BIO8), temperature seasonality (BIO4), annual mean temperature (BIO1), and mean diurnal range (BIO2) (Table 3). Based upon PI the most important variables were annual mean temperature (BIO1) and mean temperature of the direst quarter (BIO9) (Table 3). The jackknife tests of environmental variable importance for the average training gain, test gain, and test AUC showed that max temperature of warmest month (BIO5) represented the most useful information by itself, whereas omitting temperature seasonality (BIO4) had the most information not present in any of the other variables (S3B Fig). Mean diurnal range (BIO2), when used alone, produced the lowest training gain for this model.

The raster map of the average temperature model showed mainly moderate (~40%) to very high probability (85%) in Colorado, Kansas, New Mexico, Oklahoma, and Texas though areas of moderate probability occurred in other states (Fig 2B). The model also predicted an area of low probability (≤ 21%) throughout the majority of Alabama, Florida, Louisiana, and Mississippi. The temperature model also indicates an area of low probability in the Central Valley of California.

In the average full model, the highest contributing variables were maximum temperature of the warmest month (BIO5) followed by mean temperature of the wettest quarter (BIO8), mean temperature of the driest quarter (BIO9), precipitation seasonality (BIO15), and precipitation of the warmest quarter (BIO18) (Table 3). For permutation importance annual mean temperature (BIO1) and annual precipitation (BIO12) were the most important variables. Jackknifing of the average training gain, test gain, and test AUC results indicated that maximum temperature of the warmest month (BIO5) provided the most important training gain, mean diurnal range (BIO2) produced the lowest, and temperature seasonality (BIO4) had the most information that was not present in other variables (S4A Fig). The full model predicted regions of occurrences where the majority of currently known *O*. *turicata* localities occur (Fig 2C), suggesting that it may be the most accurate. A gap of probable occurrences remained between Alabama, Louisiana, and Mississippi, which were projected to be areas of very low (< 21%) to low (~21%) probability mainly along the coast. New regions of moderate probability (~43%) for the tick included arid areas of northern Mexico (Coahuila and Tamaulipas), South Carolina, and Georgia (Fig 2C).

Our average top-five model based on the percent contribution was also generated by incorporating the top-five environmental variables: maximum temperature of the warmest month (BIO5), mean temperature of the wettest quarter (BIO8), mean temperature of the driest quarter (BIO9), precipitation seasonality (BIO15), and precipitation of the warmest quarter (BIO18). The average top-five model indicated a geographical gap through Alabama, Louisiana, and Mississippi with very low (< 21%) to low (~21%) probability for *O*. *turicata* along the coast (Fig 2D). Compared to the average full model, the regions of high- to very-high probability expanded into Arizona, eastern Kansas, Oklahoma, the Texas Panhandle, and the northern Mexican states of Coahuila, Nuevo Leon, and Tamaulipas, while low probability was predicted in Georgia and South Carolina. In this model, both percent contribution and permutation importance indicated that maximum temperature of the warmest month (BIO5) had the most influence (Table 3). Jackknife test of the average training gain, test gain, and test AUC results showed that maximum temperature of the warmest month (BIO5) was the environmental variable with the highest training gain, which contained more information that was not explained by the other variables (S4B Fig). The lowest gain in this model for an individual environmental variable was precipitation seasonality (BIO15).

### Host diversity and shared geographic area

A nidicolous-ectoparasite’s distribution is not shaped exclusively by environmental variables, but it also depends heavily on host distribution, diversity, and interactions among species that are excavators, modifiers, and occupants. In historical *O*. *turicata* collections, vertebrate hosts were documented when ticks were obtained off a given animal, and the broader range of potential hosts was not considered. Therefore, we evaluated the amount of distributional overlap between likely vertebrate hosts and *O*. *turicata*. The average full model was used for the creation of the shapefile because the AUC score was the most informative (S5 Fig). Overlaying this shapefile identified the shared occupancy area between host species and *O*. *turicata*. For host species we included known and suspected vertebrate host associations derived from the USNTC database. The total area of the range of *O*. *turicata* based on this shapefile was 1,752,272 km^2^ (southeastern range = 167,318, km^2^; western range = 1,584,954 km^2^).

**Fig 3:**
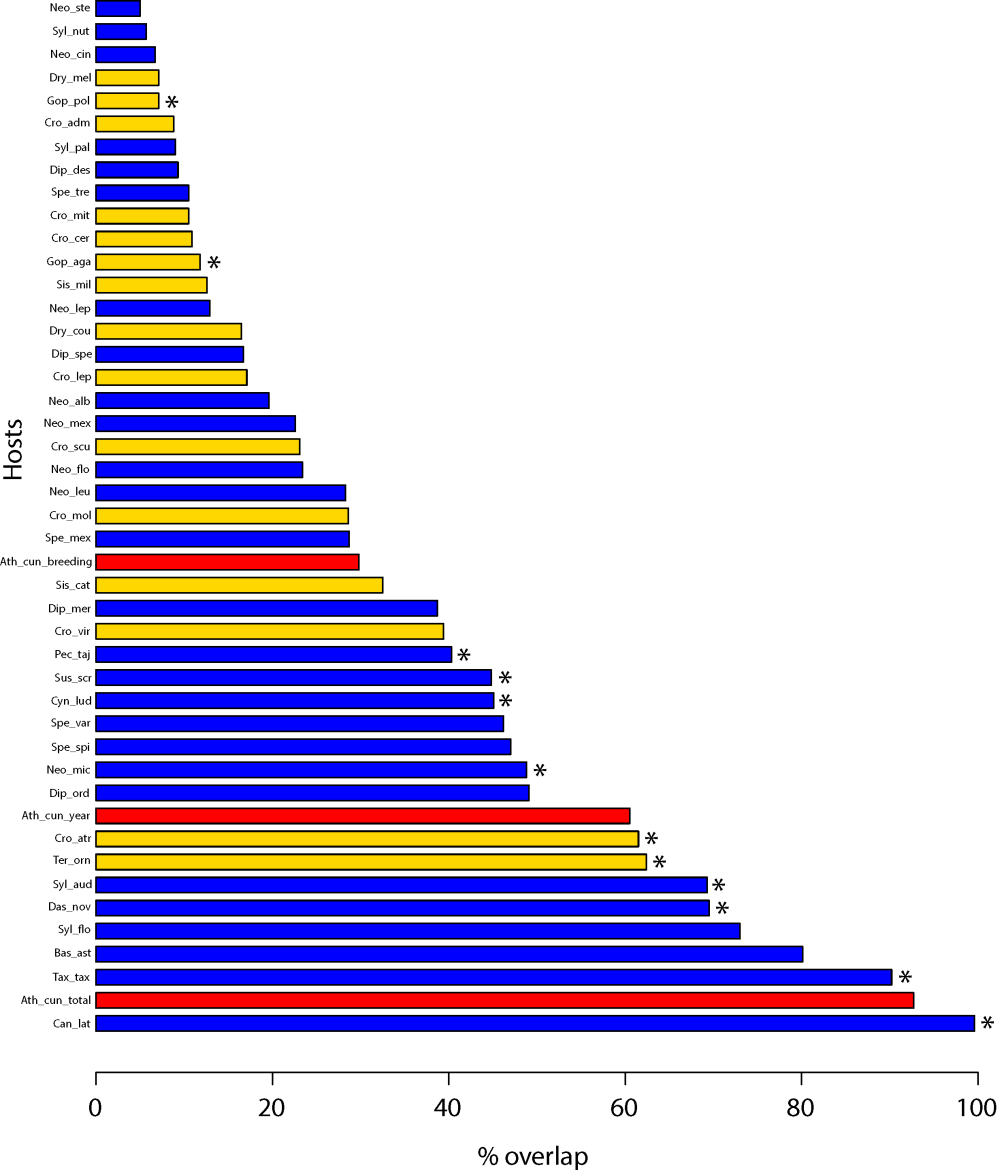
Known (asterisk) and suspected (no mark) hosts of *O*. *turicata* ranked by the percentage of their distribution overlap with the estimated range (full model, > 20% probability range = 1,752,272 km^2^) of *O*. *turicata*. Blue bars correspond to mammalian hosts, yellow bars to reptilian hosts, and the red bars to the single suspected avian host (burrowing owl, *Athene cunicularia*) whose range varies depending on their nesting phenology. Only hosts that had 5% or more overlap are included in this figure. Abbreviation code as in S3 Table where the complete list of hosts is included.

Fifty-eight known or suspected host species were found to inhabit the area within the range of *O*. *turicata*, as determined by Maxent (S3 Table). Mammalian hosts were the most numerous (*n* = 42; 72.4%), followed by reptilian (*n* = 15; 25.9%) and avian hosts were represented only by *A*. *cunicularia* (1.7%; Strigiformes, Strigidae) (Fig 3, S3 Table). The group of hosts was quite diverse as they represented a total of 8 orders, 15 families and 17 genera. Mammals comprised 5 orders, 10 families, and 11 genera, with the genus *Dipodomys* and *Neotoma* being the most diverse each with 10 species. Reptiles were represented by 2 orders, 4 families, and 5 genera with the genus *Crotalus* having the most diversity (8 species).

We assessed the overall area occupied between *O*. *turicata* and all 58 host species (S3 Table). Most (7 of 10) of the species that had half or more of their distribution overlapping with the predicted distribution of *O*. *turicata* were mammals, with the exception of two reptiles (*Terrapene ornata* and *Crotalus atrox*) and the single avian host, after considering its year-round range and total range (Fig 3). Coyotes (*C*. *latrans*) and badgers (*Taxidea taxus*), two species known as hosts of *O*. *turicata*, had a range that overlapped most of the predicted distribution of *O*. *turicata*. Ten of 12 of the host species that have been recorded to be associated with *O*. *turicata* had a range overlap of 40% or more with the predicted model, with the remaining 2 known hosts having low values (< 12%, *G*. *agassizii*, *G*. *polyphemus*,). Kangaroo rats (*Dipodomys*) represent one of the most diverse genera of hosts for *O*. *turicata* but most of them had limited distributions and thus low overlaps (< 10%), except for two species, *D*. *ordii* and *D*. *merriami*, which had moderate to large overlaps, 49.1 and 38.7%, respectively. Rattlesnakes (*Crotalus*) were the most diverse genus of reptilian hosts showing low (8–10%) to large (61.5%) range overlap with the *O*. *turicata* distribution model.

## Discussion

This study utilized database searches, field collections, and a maximum entropy modeling approach to generate a present-day geographic distribution prediction of localities for *O*. *turicata* in the United States and north Mexico. A stringent definition was used to generate georeferenced data points, potentially excluding regions of Latin America where *O*. *turicata* may exist. For example, recent case reports have described human infection to relapsing fever spirochetes in Sonora, Mexico and along the Guatemala-Belize border [56,57]. However, the reports did not present molecular evidence of *B*. *turicatae* infection, nor were ticks collected at the presumed exposure sites, and thus these localities were excluded from our analyses. Additionally, Dr. Oscar Felsenfeld described *O*. *turicata* into South America [2], yet to our knowledge ticks were not collected or morphological features noted to speciate the vectors and the region was omitted. These reports highlight the need to expand research efforts to understand the distribution of *O*. *turicata* in the Neotropics, a region where the vectors and pathogens they transmit have been overlooked.

The nidiculous life cycle and rapid feeding behavior of *O*. *turicata* has resulted in the collection of few specimens, and our understanding of the tick’s distribution is limited. A total of 158 *O*. *turicata* localities were obtained for this study, and over 90 collections occurred prior to 1950. Moreover, habitat information for ticks was sparse and solely at the county level and estimated georeferenced localities. Consequently, additional fine scale studies were not possible. Regardless, the Maxent analyses in this report have provided the framework to initiate field studies to further define the habitat of *O*. *turicata*. We envision microecological analyses of localities where ticks have been collected to better define burrow communities.

From the four SDMs generated, we hypothesize that the models including all and the top-five environmental variables most accurately predict the distribution of *O*. *turicata*. The model that included only the top-five variables predicted the Florida Panhandle and central California as a region of low to very low probability for *O*. *turicata*. While we are unaware of current day field collections in central California, we recently obtained *O*. *turicata* from gopher tortoise dens as far west in Florida as Apalachicola National Forest, which indicates that the Florida Panhandle is an ecologically suitable region for this tick species.

The models that exclusively used temperature or precipitation variables are likely the least accurate. The precipitation model predicted ranges of very low to low probability for *O*. *turicata* in regions where ticks have recently been collected, such as the Florida Panhandle and Joshua Tree National Park, California [58]. Additionally, the temperature model may inaccurately predict regions of probable occurrences throughout Alabama, Louisiana, and Mississippi, where a present day gap exists. While Maxent requires presence only data, an extensive evaluation of gopher tortoise dens in Mississippi failed to identify soft ticks [46], and supports prediction models that suggest most of this region may not sustain *O*. *turicata*.

**Fig 4:**
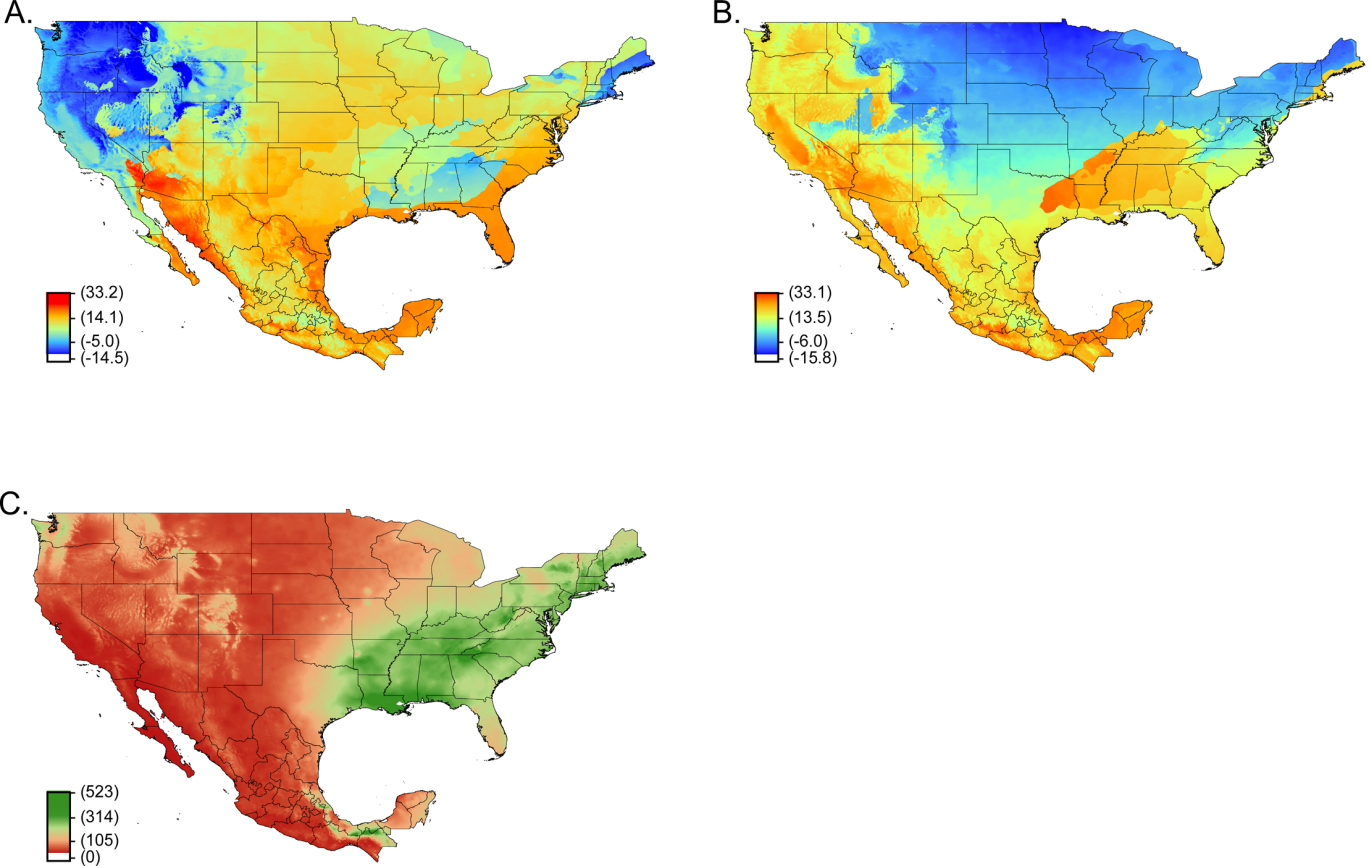
Environmental variables that may explain the gap between populations of *O*. *turicata* in Florida and Texas. Shown are mean temperature of the wettest quarter (BIO8) (A), mean temperature of driest quarter (BIO9) (B), and precipitation of the driest quarter (BIO17) (C). The gradient of red to blue represents high to low temperatures (°C) (A–B). For the precipitation variable, a gradient of green to dark brown represents the amount of high to low precipitation (mm) (C).

**Fig 5:**
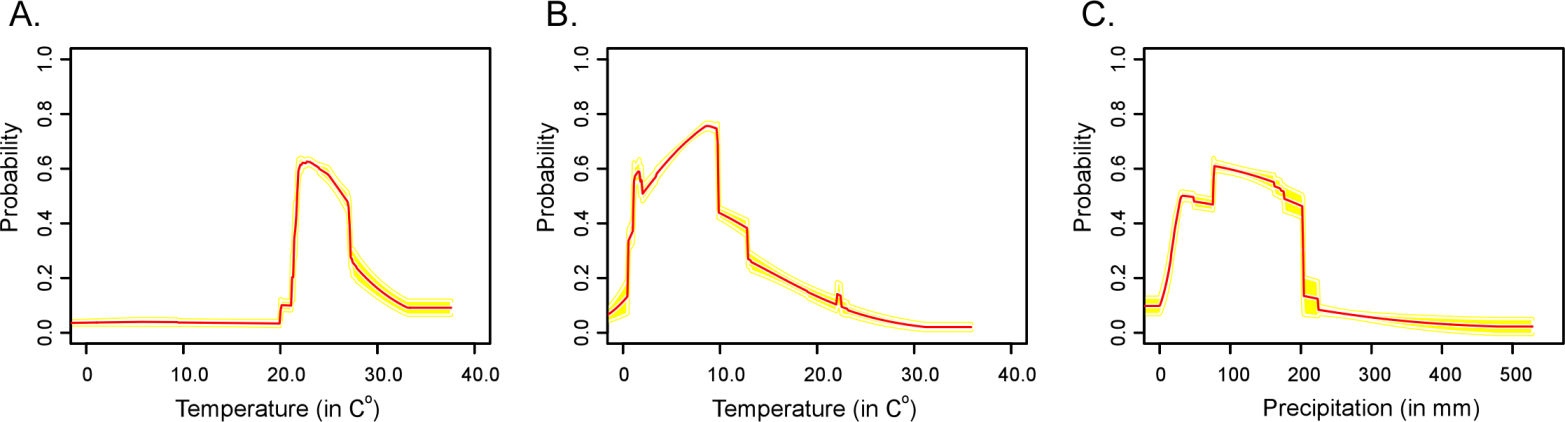
Three response curves with only corresponding variables in the *O*. *turicata* model: A) mean temperature of the wettest quarter (BIO8), B) mean temperature of the driest quarter (BIO9), and C) precipitation of the driest quarter (BIO17). Mean response of 10 Maxent runs (red) and the mean ± 1 standard deviation (yellow).

The geographical gap between Texas and Florida may occur due to unique current climate conditions associated with this geographic area. Evaluating the original environmental variable inputs used in the Maxent models, this gap area may be a result of low temperatures during the wettest quarter (BIO8) (Fig 4A) and high temperatures during the driest quarter (BIO9) (Fig 4B). The response curves for BIO8 showed that low probability is stable until temperatures reach 20°C where probability started to increase then declined (Fig 5A). While the response curve for BIO9 indicated that the probability increased with low temperatures, it then declined at higher temperatures (Fig 5B). In addition, this gap area also receives greater than 200 mm of precipitation during the driest quarter (Fig 4C). Evaluating the response curve with BIO17 as a variable (Fig 5C) the probability drops off at 200 mm within this gap region. The precipitation and temperature variables may explain the gap occurring between Texas and Florida, but whether this vicariance event was due to recent changes in the environment is unknown.

Another possibility for the disjunction between Texas and Florida is an extinction event in the intervening gap area, or the introduction of *O*. *turicata* by long-distance dispersal of a migratory animal. Currently, burrowing owl populations in Florida are predominately year-round residents [59] and are distinct from migrating western burrowing owls [60]. Interestingly, our work in Texas identified nymphal *Carios kelleyi*, a genus of soft ticks that also rapidly feed as nymphs and adults, on bats as they emerged from the roost. Given similar collections of *O*. *turicata* from caves and the indiscriminant feeding behavior of these ticks, bats may have an important role in soft tick dispersal. Clearly, much is unknown regarding the mechanistic explanation of this distribution gap, and further phylogeographic and modeling studies will provide insight toward gene flow between the populations of *O*. *turicata*.

To comprehend the dispersal of *O*. *turicata* and define suitable habitat for the ticks, field studies and additional tick collections are needed throughout the United States. Our study identified new regions to focus upon, including portions of Georgia, North and South Carolina, Kansas, Oklahoma, and Nevada. Moreover, given the geographical overlap of *O*. *turicata* with *O*. *parkeri* and *O*. *talaje*, do the two species of tick share similar habitat and if so does the host community support the fitness of one tick species over another?

The peculiar life-cycle of soft ticks presents unique challenges for understanding their biology, distributions, and the ecology of the pathogens they transmit. In general the ticks are rapid nocturnal feeders and most of their life-cycle occurs in a nest, den, or cave, which serve as a micro-refuge from extreme temperatures, humidity, fire, and predation [61]. Also, they are rarely encountered on the vertebrate host. Therefore, the identification of endemic regions can be difficult and requires the development of non-traditional tick surveillance methods, such as serological assays to detect antibodies against genus or species-specific tick salivary antigens [62–64].

In this report, we included a total of 58 vertebrate host species, but because burrows are frequently in a state of flux with primary excavators, modifiers, and occupants, there are likely more potential hosts. Bloodmeal sources not included in the study encompass species of rodents such as voles (*Microtus*), mice (*Mus*, *Onychomys*, and *Peromyscus*), cotton rats (*Sigmodon*), and gophers (*Geomys*). Other potential vertebrate hosts include members of Mustelidae, small mammals (badgers, ferrets, and weasels) that dwell in habitat utilized by burrowing owls [65,66]. Reptilian burrow occupants such as racers (*Coluber*) and the gopher snake (*Pituophis catenifer*) should also be evaluated as potential hosts for *O*. *turicata* in addition to lizards that reside in burrows and outcroppings.

The complex host community composition (species makeup) and structure (relative abundance) at a given site will likely influence the ecology of soft tick populations and the pathogens they transmit. Within the list of known hosts, several species have a high spatial overlap, as predicted by our model, and are prevalent components of local communities. The indiscriminant feeding behavior of *Ornithodoros* species and diversity of vertebrate hosts increases the likelihood of supporting the life-cycle of soft ticks. However, less understood is the host’s role in maintaining pathogens transmitted by soft ticks.

*Ornithodoros* species are important vectors of human and veterinary pathogens [19,30,33,67]. Studies suggest that in addition to vertebrates, the ticks could be a reservoir for microorganisms given their long life-span and ability to endure prolonged periods of starvation [20]. *Borrellia turicatae*, a human and canine pathogen, is maintained throughout the life-cycle of *O*. *turicata* [1,30,33], and in rodents a full bloodmeal by the ticks is not required for establishing infection [68]. An important step toward understanding the ecology of this pathogen would be to ascertain which additional vertebrate hosts help maintain *B*. *turicatae* in nature. Studies in *Borrelia burgdorferi* indicate that within an ecological setting, community composition, host diversity and competency for the pathogens has profound implications for the risk of Lyme disease in human populations [69]. Presumably, there are patterns that govern the transmission and maintenance of *B*. *turicatae*, but the ecology of this pathogen remains vague.

*Ornithodoros turicata* is also one of several North American species found capable of transmitting ASFV [6,8,70]. ASFV is an ever-present biosecurity concern to domestic swine industries in the Western Hemisphere, and has been spreading globally over the last decade [7]. The Global Alliance for ASF has emerged to address surveillance, prevention, control, and research needs [71] as the virus inflicts severe economic damage and causes nearly 100% mortality in susceptible domestic pig populations [7,72]. The expansion and ecological overlap of feral swine and *O*. *turicata* in the southern United States, and the longevity and feeding behavior of this soft tick has increased the risk for the emergence of ASFV in North America [73]. Our predictive SDMs will guide future studies to understand the ecology and increase awareness of pathogens transmitted by *O*. *turicata*.

## Supporting Information

S1 Fig Response curves with all variables (BIO1-19 and ALT) in the *O*. *turicata* model show the mean response of the 10 Maxent runs (red) and the mean ± 1 standard deviation (yellow). (PDF)

S2 Fig Response curves with only each corresponding variable in the *O*. *turicata* model show the mean response for 10 Maxent runs (red) and the mean ± 1 standard deviation (yellow). (PDF)

S3 Fig Average jackknife results of the training gain, tests gain, and test AUC for *O*. *turicata*: A) precipitation model (top three panels); and B) temperature model (lower three panels). Red bar indicates training gain of all variables in the model, while blue represents the training gain for including only a single variable in the model, and yellow is the exclusion of variable.
(PDF)

S4 Fig Average jackknife results of the training gain, tests gain, and test AUC for *O*. *turicata*: A) full model (top three); and B) top-five environmental variables model (lower three). Red bar indicates training gain of all variables in the model, while blue represents the training gain for including only a single variable in the model, and yellow is the exclusion of variable.
(PDF)

S5 Fig The shapefile of the full model based on the most informative AUC score, which was used as an overlay to determine shared occupancy between known and potential host species and *O*. *turicata*. The dark outline depicts the predicted distribution of *O*. *turicata* and black dots represent localities of tick collections.
(PDF)

S1 Table Twenty environmental layers with variable code acquired from the WorldClim dataset (http://worldclim.org). (PDF)

S2 Table Correlation matrix of all twenty environmental layers. Positive correlation ≥ 0.80 shown in red and negative correlation ≤ -0.80 shown in yellow. For variable names refer to the S1 Table.
(PDF)

S3 Table List of known and suspected host species of *O*. *turicata*. For each species, its estimated distribution in the United States and Mexico, the calculated area shared with *O*. *turicata* and what percentage this represents from the estimated soft tick range (> 20% probability range = 1,752,272 km^2^) are included.
(PDF)

S4 Table Literature search of tick-borne relapsing fever spirochetes as an indicator of *O*. *turicata* distribution. (PDF)
