# Correction: Assessment of the Geographic Distribution of *Ornithodoros turicata* (Argasidae): Climate Variation and Host Diversity

**DOI:** 10.1371/journal.pntd.0004538

**Published:** 2016-03-08

**Authors:** Taylor G. Donaldson, Adalberto A Pèrez de León, Andrew Y. Li, Ivan Castro-Arellano, Edward Wozniak, William K. Boyle, Reid Hargrove, Hannah K. Wilder, Hee J. Kim, Pete D. Teel, Job E. Lopez

The third author’s middle initial is listed incorrectly. The correct name is Andrew Y. Li. The correct citation is: Donaldson TG, Pèrez de León AA, Li AY, Castro-Arellano I, Wozniak E, Boyle WK, et al. (2016) Assessment of the Geographic Distribution of *Ornithodoros turicata* (Argasidae): Climate Variation and Host Diversity. PLoS Negl Trop Dis 10(2): e0004383. doi:10.1371/journal.pntd.0004383
