# Supplementary figures and images for: Assessment of the Geographic Distribution of Ornithodoros turicata (Argasidae): Climate Variation and Host Diversity
Source: PLoS Negl Trop Dis. 2016 Feb 1;10(2):e0004383. doi: 10.1371/journal.pntd.0004383 (PMC4734830; doi:10.1371/journal.pntd.0004383)

**Bio1**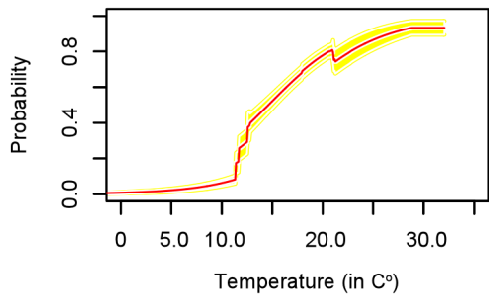**Bio2**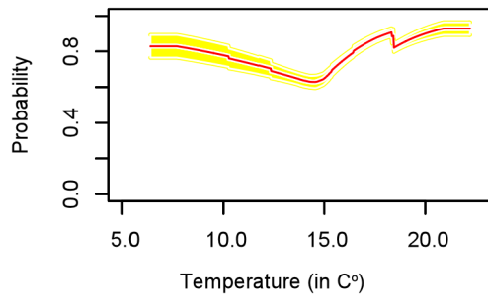**Bio4**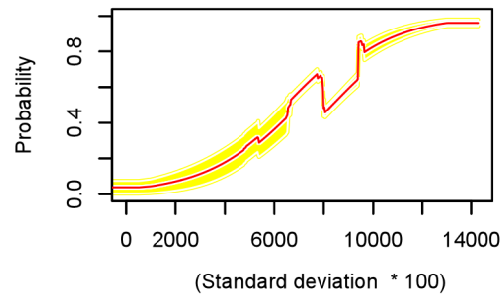**Bio5**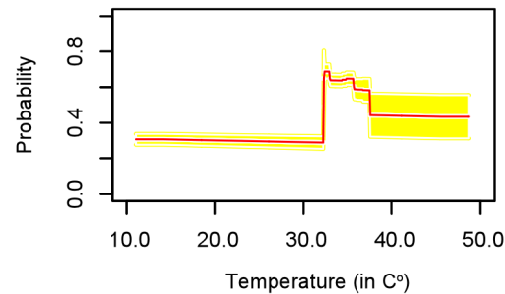**Bio8**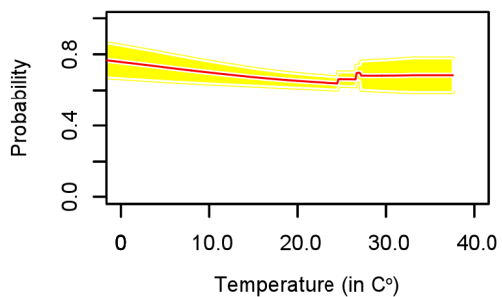**Bio9**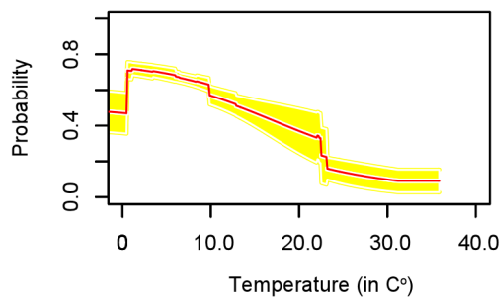**Bio12**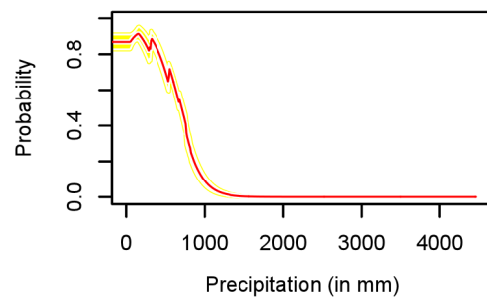**Bio15**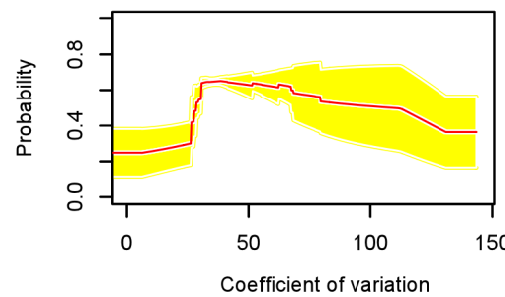**Bio17**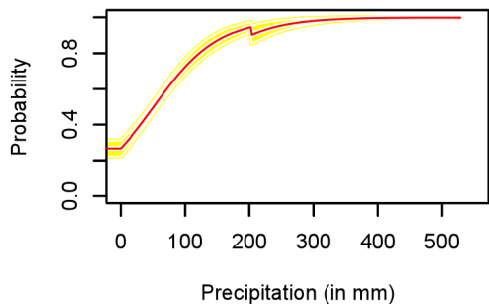**Bio18**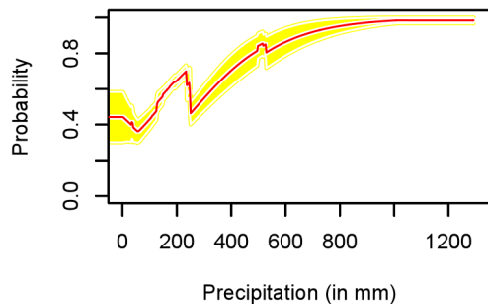**Bio19**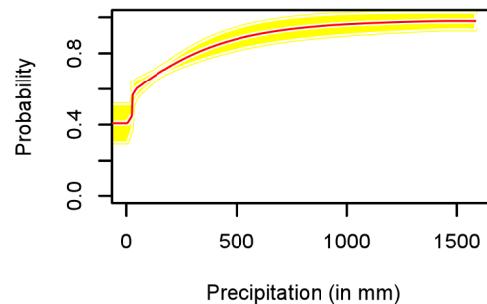**ALT**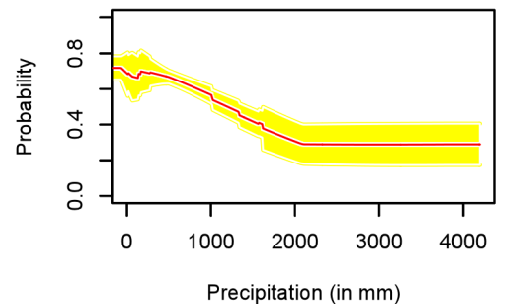

Supplement: S1 Fig — (PDF) [file pntd.0004383.s001.pdf]

**Bio1**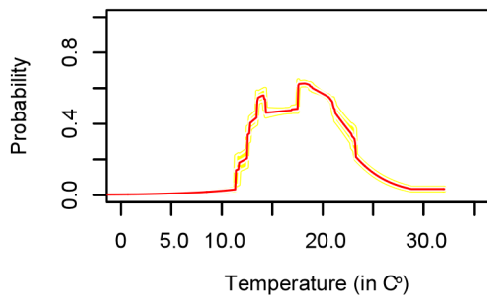**Bio2**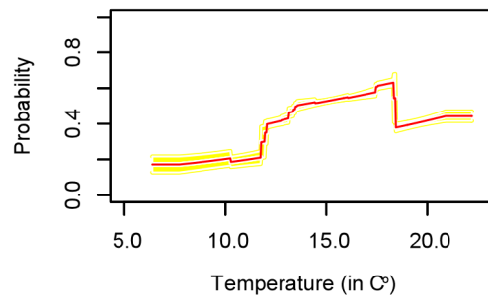**Bio4**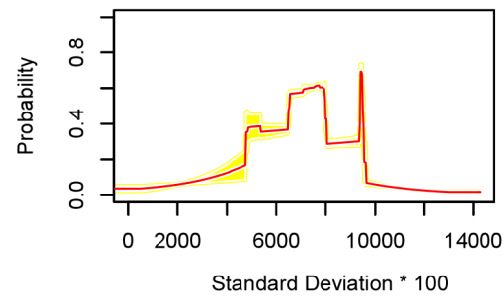**Bio5**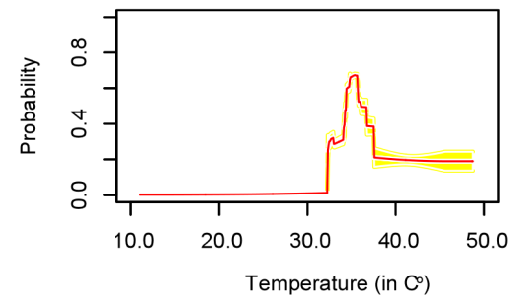**Bio8**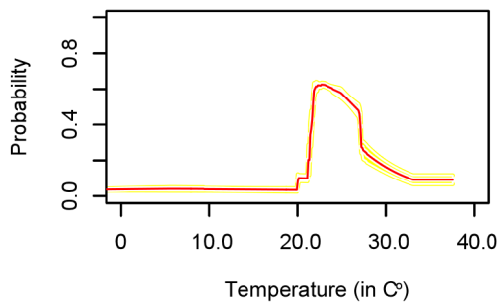**Bio9**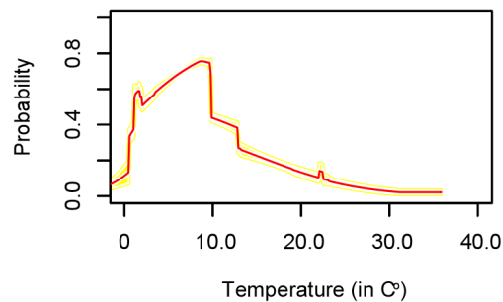**Bio12**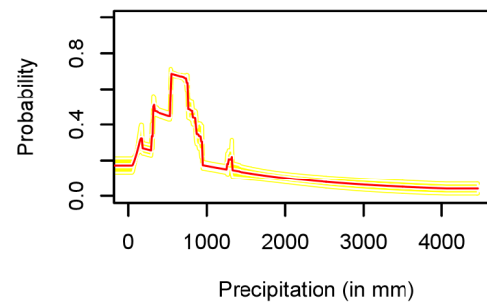**Bio15**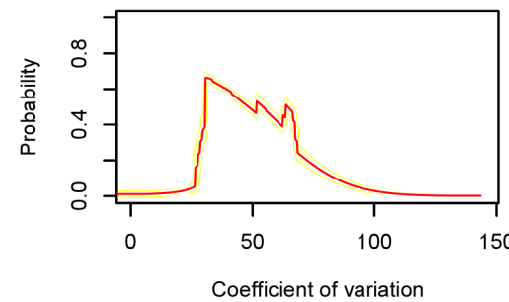**Bio17**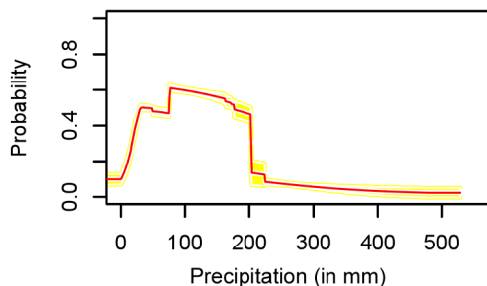**Bio18**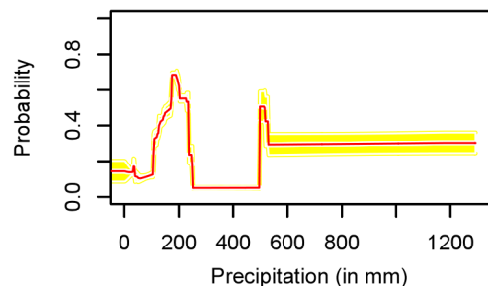**Bio19**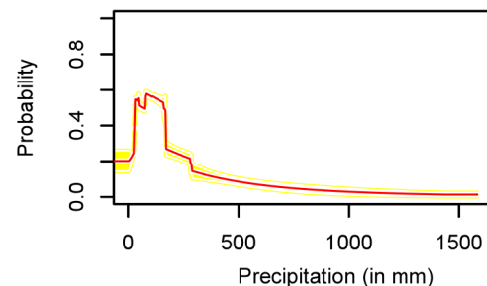**ALT**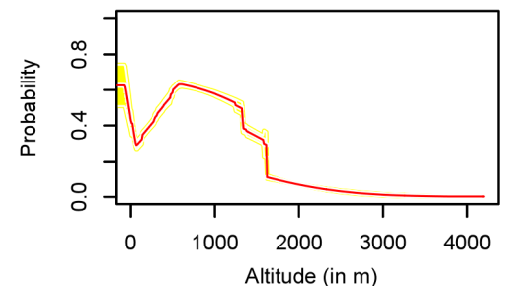

Supplement: S2 Fig — (PDF) [file pntd.0004383.s002.pdf]

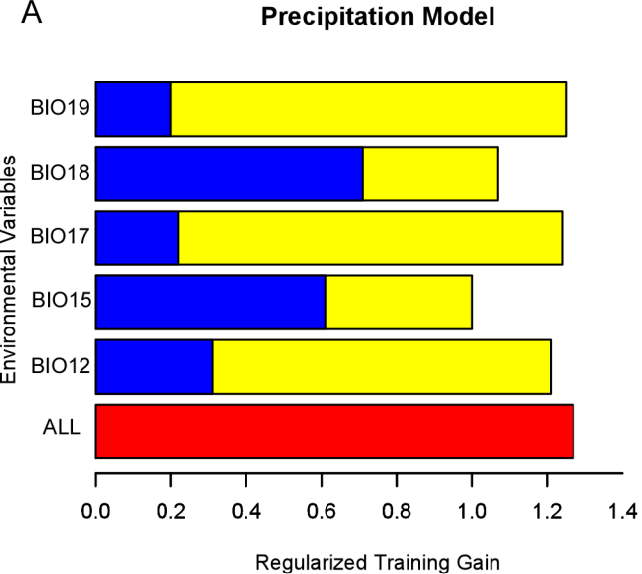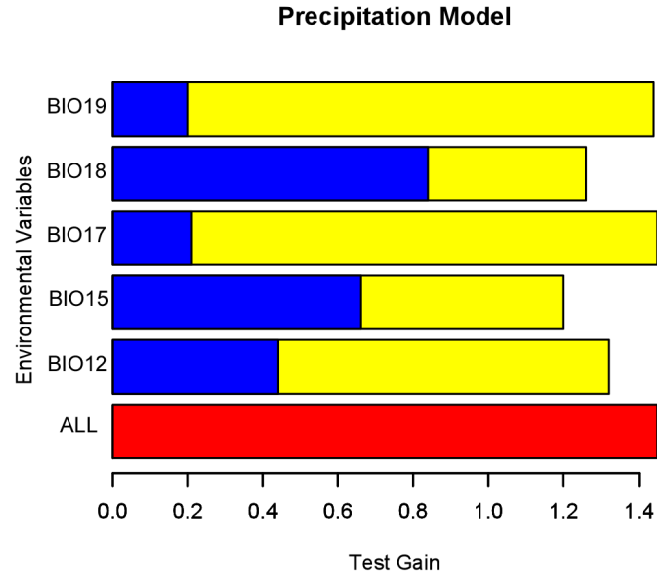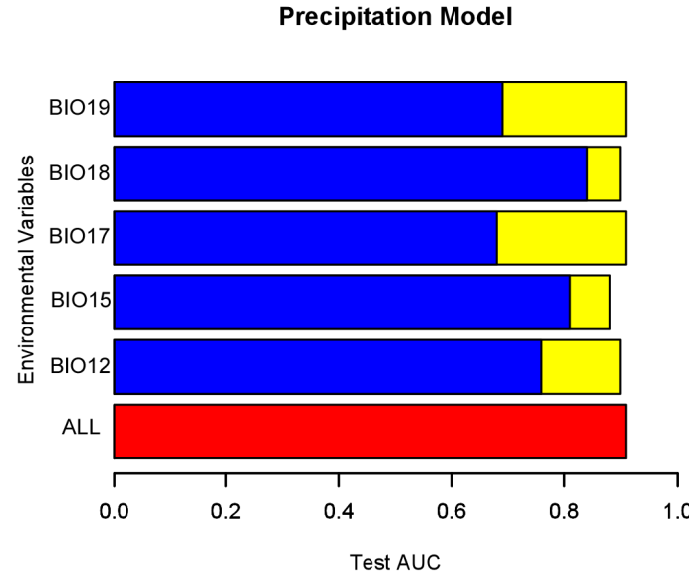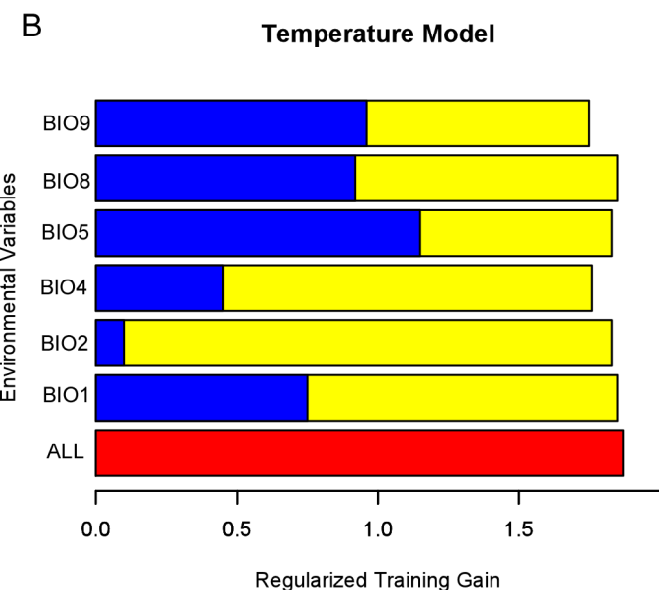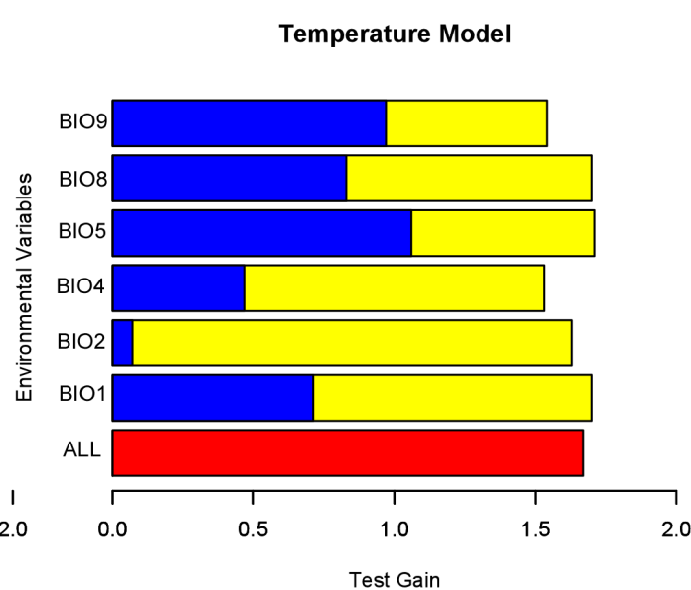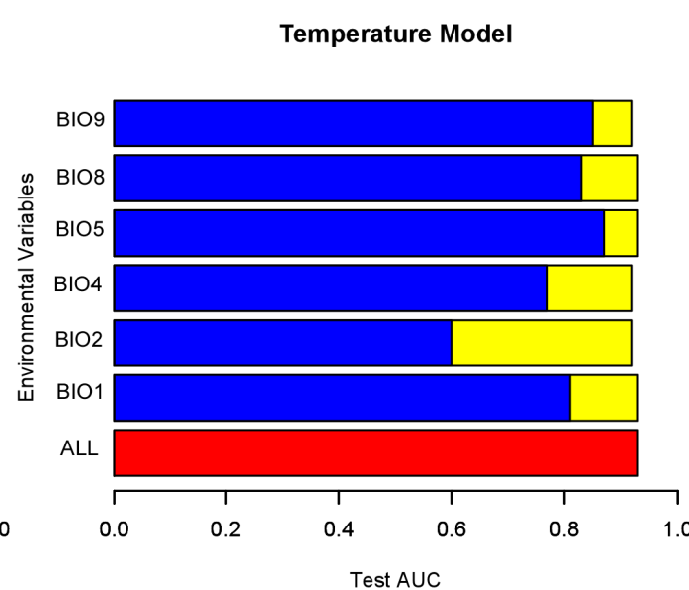

Supplement: S3 Fig — Red bar indicates training gain of all variables in the model, while blue represents the training gain for including only a single variable in the model, and yellow is the exclusion of variable. (PDF) [file pntd.0004383.s003.pdf]

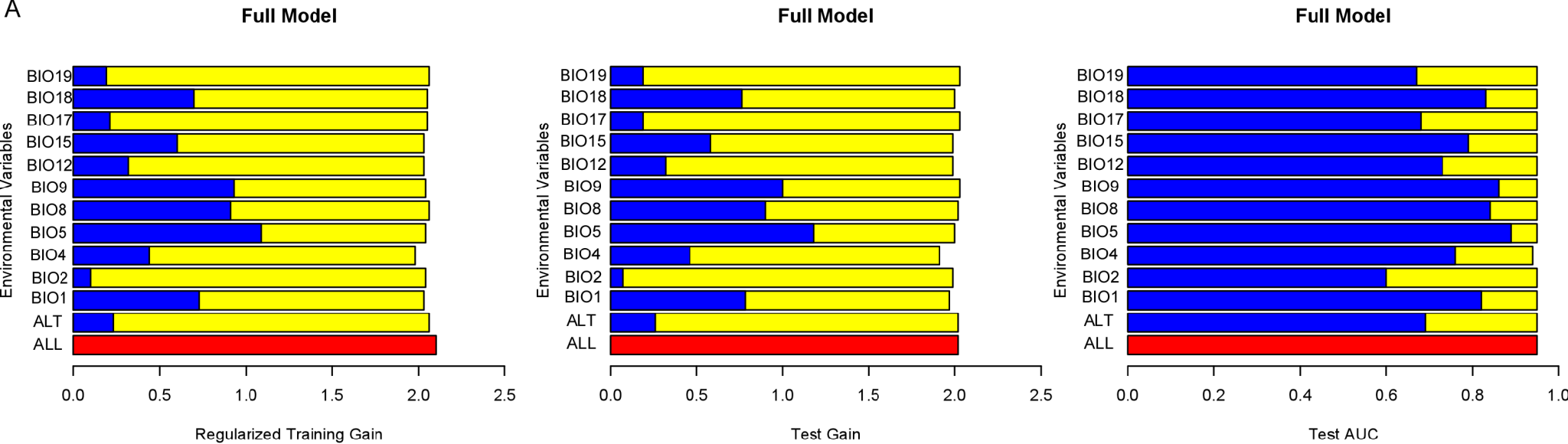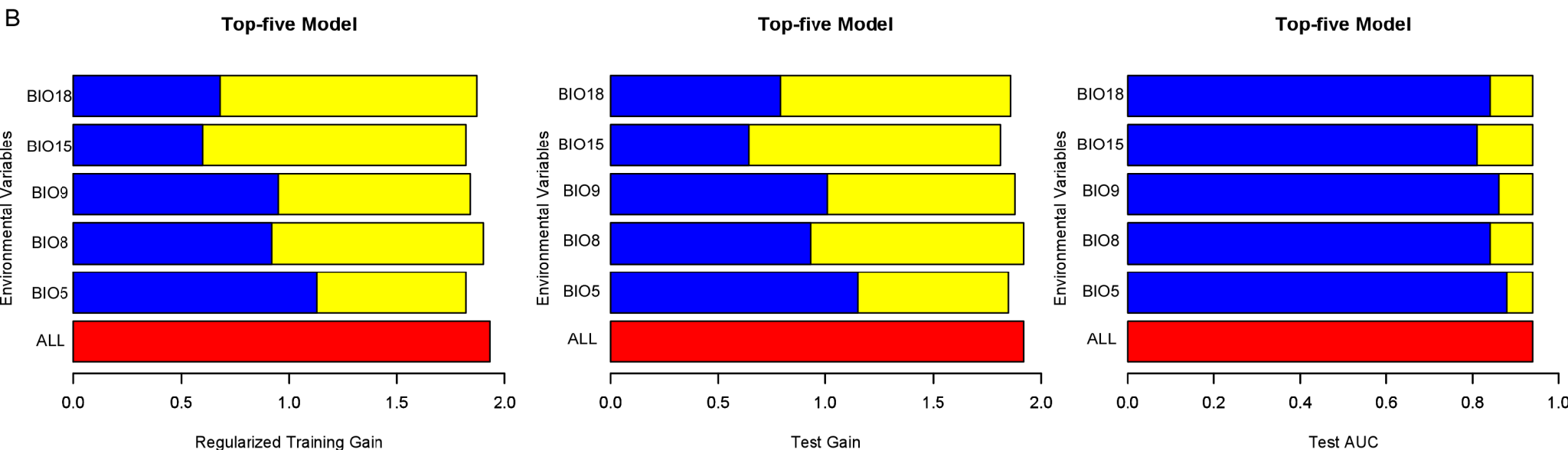

Supplement: S4 Fig — Red bar indicates training gain of all variables in the model, while blue represents the training gain for including only a single variable in the model, and yellow is the exclusion of variable. (PDF) [file pntd.0004383.s004.pdf]

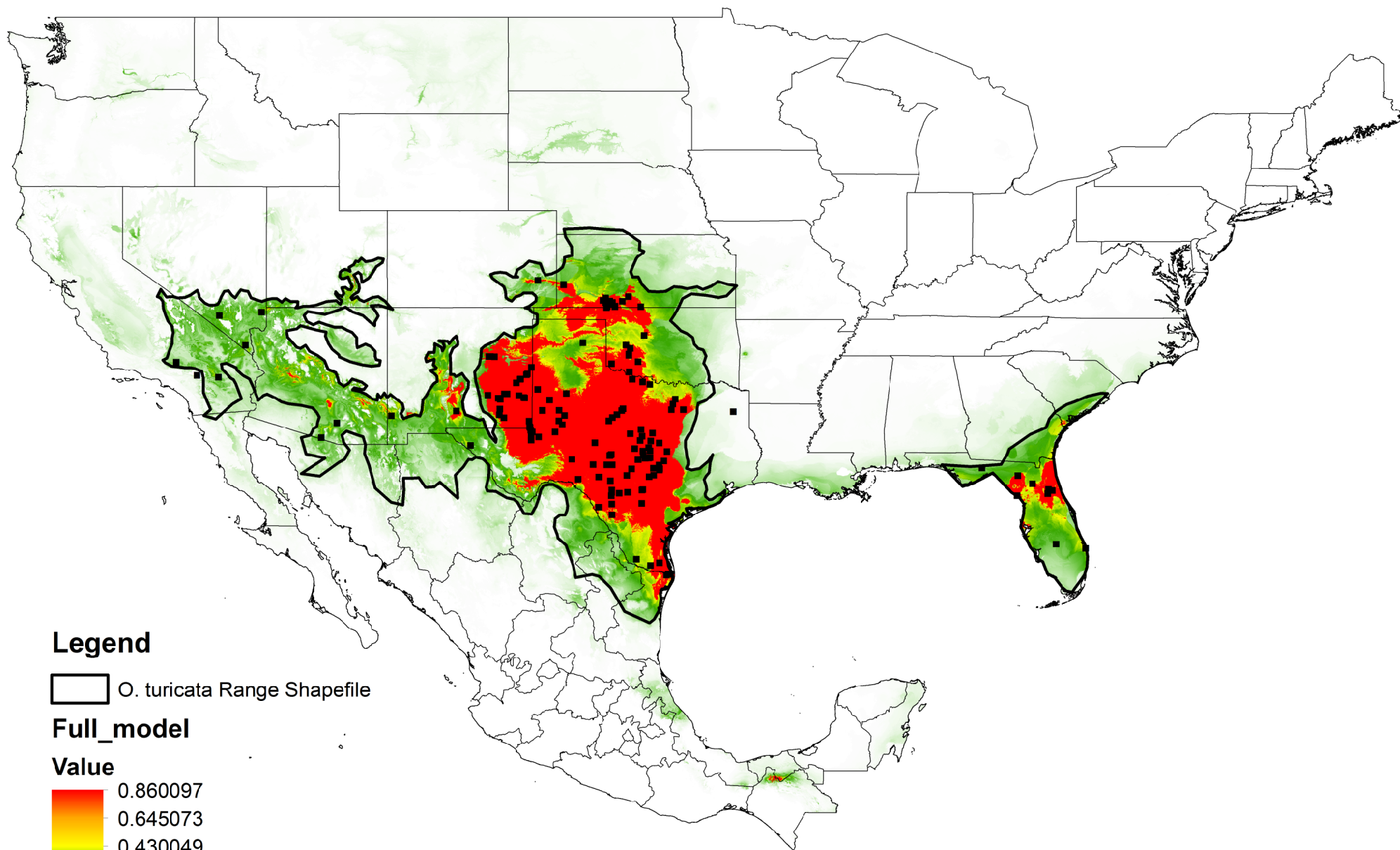

## Legend

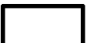 *O. turicata* Range Shapefile

## Full\_model

### Value

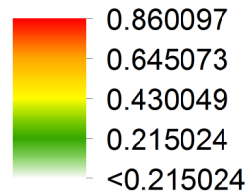

Supplement: S5 Fig — The dark outline depicts the predicted distribution of O. turicata and black dots represent localities of tick collections. (PDF) [file pntd.0004383.s005.pdf]
