# Supplementary material for: Assessment of the Geographic Distribution of Ornithodoros turicata (Argasidae): Climate Variation and Host Diversity
Source: PLoS Negl Trop Dis. 2016 Feb 1;10(2):e0004383. doi: 10.1371/journal.pntd.0004383 (PMC4734830; doi:10.1371/journal.pntd.0004383)
Supplement: S1 Table — (PDF) [file pntd.0004383.s006.pdf]

S1 Table. Twenty environmental layers with variable code acquired from the WorldClim dataset (http://worldclim.org).

Variable Code	Variable Name
BIO1	Annual Mean Temperature
BIO2	Mean Diurnal Range
BIO3	Isothermality
BIO4	Temperature Seasonality
BIO5	Maximum Temperature of Warmest Month
BIO6	Minimum Temperature of Coldest Month
BIO7	Temperature Annual Range
BIO8	Mean Temperature of Wettest Quarter
BIO9	Mean Temperature of Driest Quarter
BIO10	Mean Temperature of Warmest Quarter
BIO11	Mean Temperature of Coldest Quarter
BIO12	Annual Precipitation
BIO13	Precipitation of Wettest Month
BIO14	Precipitation of Driest Month
BIO15	Precipitation Seasonality
BIO16	Precipitation of Wettest Quarter
BIO17	Precipitation of Driest Quarter
BIO18	Precipitation of Warmest Quarter
BIO19	Precipitation of Coldest Quarter
ALT	Altitude
