# Supplementary material for: Assessment of the Geographic Distribution of Ornithodoros turicata (Argasidae): Climate Variation and Host Diversity
Source: PLoS Negl Trop Dis. 2016 Feb 1;10(2):e0004383. doi: 10.1371/journal.pntd.0004383 (PMC4734830; doi:10.1371/journal.pntd.0004383)
Supplement: S2 Table — Positive correlation ≥ 0.80 shown in red and negative correlation ≤ -0.80 shown in yellow. For variable names refer to the S1 Table. (PDF) [file pntd.0004383.s007.pdf]

S2 Table. Correlation matrix of all twenty environmental layers. Positive correlations ≥ 0.80 shown in red and negative correlations ≤ -0.80 shown in yellow. For variable names refer to the S1 Table.

	BIO1	BIO2	BIO3	BIO4	BIO5	BIO6	BIO7	BIO8	BIO9	BIO10	BIO11	BIO12	BIO13	BIO14	BIO15	BIO16	BIO17	BIO18	BIO19	ALT
**BIO1**	**1.00000**	0.03370	0.65141	-0.69035	0.79086	0.93891	-0.70876	0.60665	0.71150	0.87390	0.95602	0.24687	0.40067	0.03689	0.36658	0.35446	0.04674	0.31015	0.02941	-0.36906
**BIO2**	0.03370	**1.00000**	0.40684	-0.14158	0.29461	-0.01004	0.17556	-0.04022	0.17225	-0.01699	0.09784	-0.63989	-0.42746	-0.55585	0.38066	-0.45303	-0.57517	-0.48835	-0.46021	0.62933
**BIO3**	0.65141	0.40684	**1.00000**	-0.93627	0.29998	0.78439	-0.79205	0.19534	0.68091	0.24988	0.81945	0.05089	0.38452	-0.30020	0.61371	0.34662	-0.29505	0.05929	-0.05520	0.29948
**BIO4**	-0.69035	-0.14158	-0.93627	**1.00000**	-0.22366	-0.87370	0.94347	-0.13987	-0.75597	-0.25561	-0.86990	-0.26242	-0.51638	0.08915	-0.46215	-0.48523	0.07317	-0.15751	-0.17455	-0.16254
**BIO5**	0.79086	0.29461	0.29998	-0.22366	**1.00000**	0.59059	-0.16730	0.57133	0.48816	0.92644	0.63876	-0.13294	-0.03067	-0.13834	0.25473	-0.07573	-0.14141	0.00151	-0.24002	-0.37143
**BIO6**	0.93891	-0.01004	0.78439	-0.87370	0.59059	**1.00000**	-0.89440	0.40757	0.81298	0.67318	0.98992	0.32514	0.50551	0.03680	0.37636	0.46649	0.05332	0.26292	0.17611	-0.22818
**BIO7**	-0.70876	0.17556	-0.79205	0.94347	-0.16730	-0.89440	**1.00000**	-0.18128	-0.72268	-0.30898	-0.85539	-0.47092	-0.63459	-0.12164	-0.31862	-0.61190	-0.14351	-0.32038	-0.34819	0.07291
**BIO8**	0.60665	-0.04022	0.19534	-0.13987	0.57133	0.40757	-0.18128	**1.00000**	0.00907	0.69790	0.45754	0.06166	0.18294	-0.04753	0.32089	0.14436	-0.05321	0.43497	-0.31937	-0.37156
**BIO9**	0.71150	0.17225	0.68091	-0.75597	0.48816	0.81298	-0.72268	0.00907	**1.00000**	0.46371	0.79830	0.19217	0.29076	0.04989	0.20017	0.26736	0.06703	-0.01957	0.29401	-0.04413
**BIO10**	0.87390	-0.01699	0.24988	-0.25561	0.92644	0.67318	-0.30898	0.69790	0.46371	**1.00000**	0.69892	0.12903	0.16548	0.09272	0.17737	0.12489	0.09459	0.26860	-0.07952	-0.58264
**BIO11**	0.95602	0.09784	0.81945	-0.86990	0.63876	0.98992	-0.85539	0.45754	0.79830	0.69892	**1.00000**	0.25611	0.46334	-0.02220	0.43367	0.41939	-0.00915	0.24913	0.08968	-0.17480
**BIO12**	0.24687	-0.63989	0.05089	-0.26242	-0.13294	0.32514	-0.47092	0.06166	0.19217	0.12903	0.25611	**1.00000**	0.85071	0.72049	-0.24754	0.87344	0.74870	0.72423	0.75516	-0.46323
**BIO13**	0.40067	-0.42746	0.38452	-0.51638	-0.03067	0.50551	-0.63459	0.18294	0.29076	0.16548	0.46334	0.85071	**1.00000**	0.29678	0.24202	0.99124	0.32714	0.67797	0.53181	-0.27692
**BIO14**	0.03689	-0.55585	-0.30020	0.08915	-0.13834	0.03680	-0.12164	-0.04753	0.04989	0.09272	-0.02220	0.72049	0.29678	**1.00000**	-0.71997	0.32393	0.99267	0.53931	0.62216	-0.45948
**BIO15**	0.36658	0.38066	0.61371	-0.46215	0.25473	0.37636	-0.31862	0.32089	0.20017	0.17737	0.43367	-0.24754	0.24202	-0.71997	**1.00000**	0.20405	-0.71706	-0.05150	-0.34308	0.21100
**BIO16**	0.35446	-0.45303	0.34662	-0.48523	-0.07573	0.46649	-0.61190	0.14436	0.26736	0.12489	0.41939	0.87344	0.99124	0.32393	0.20405	**1.00000**	0.35506	0.67451	0.57226	-0.28296
**BIO17**	0.04674	-0.57517	-0.29505	0.07317	-0.14141	0.05332	-0.14351	-0.05321	0.06703	0.09459	-0.00915	0.74870	0.32714	0.99267	-0.71706	0.35506	**1.00000**	0.54648	0.66016	-0.46924
**BIO18**	0.31015	-0.48835	0.05929	-0.15751	0.00151	0.26292	-0.32038	0.43497	-0.01957	0.26860	0.24913	0.72423	0.67797	0.53931	-0.05150	0.67451	0.54648	**1.00000**	0.22742	-0.42103
**BIO19**	0.02941	-0.46021	-0.05520	-0.17455	-0.24002	0.17611	-0.34819	-0.31937	0.29401	-0.07952	0.08968	0.75516	0.53181	0.62216	-0.34308	0.57226	0.66016	0.22742	**1.00000**	-0.31054
**ALT**	-0.36906	0.62933	0.29948	-0.16254	-0.37143	-0.22818	0.07291	-0.37156	-0.04413	-0.58264	-0.17480	-0.46323	-0.27692	-0.45948	0.21100	-0.28296	-0.46924	-0.42103	-0.31054	**1.00000**
