# Supplementary material for: Assessment of the Geographic Distribution of Ornithodoros turicata (Argasidae): Climate Variation and Host Diversity
Source: PLoS Negl Trop Dis. 2016 Feb 1;10(2):e0004383. doi: 10.1371/journal.pntd.0004383 (PMC4734830; doi:10.1371/journal.pntd.0004383)
Supplement: S3 Table — For each species, its estimated distribution in the United States and Mexico, the calculated area shared with O. turicata and what percentage this represents from the estimated soft tick range (> 20% probability range = 1,752,272 km2) are included. (PDF) [file pntd.0004383.s008.pdf]

S3 Table. List of known and suspected host species of *Ornithodoros turicata*. For each species its estimated distribution in the United States and Mexico, the calculated area shared with *O. turicata* and what percentage this represents from the estimated soft tick range (> 20% probability range = 1,752,272 km²) are included.

Class	Order	Family	Species	Common Name	Species Code	Host Status	Area of Known Distribution (km²)	Area of Shared Occupancy (km²)	Percentage of Area Shared Occupancy
Aves	Strigiformes	Strigidae	*Athene cunicularia*	Burrowing Owl	Ath_cun_total	Suspected	5,798,608	1,624,015	92.7
Aves	Strigiformes	Strigidae	*Athene cunicularia*	Burrowing Owl	Ath_cun_breeding	Suspected	2,900,512	499,893	29.8
Aves	Strigiformes	Strigidae	*Athene cunicularia*	Burrowing Owl	Ath_cun_non_breeding	Suspected	770,189	64,385	3.7
Aves	Strigiformes	Strigidae	*Athene cunicularia*	Burrowing Owl	Ath_cun_year	Suspected	2,127,907	1,059,737	60.5
Mammalia	Artiodactyla	Suidae	*Sus scrofa*	Feral Swine	Sus_scr	Known	1,580,579	769,993	44.8
Mammalia	Artiodactyla	Tayassuidae	*Pecari tajacu*	Collard Peccary	Pec_taj	Known	1,657,135	706,207	40.3
Mammalia	Carnivora	Canidae	*Canis latrans*	Coyote	Can_lat	Known	10,989,479	1,746,024	99.6
Mammalia	Carnivora	Mustelidae	*Taxidea taxus*	American Badger	Tax_tax	Known	7,021,927	1,580,357	90.2
Mammalia	Carnivora	Procyonidae	*Bassariscus astutus*	Ringtail	Bas_ast	Suspected	5,429,236	1,402,978	80.1
Mammalia	Cingulata	Dasypodidae	*Dasypus novemcinctus*	Nine-banded Armadillo	Das_nov	Known	3,654,165	1,214,442	69.5
Mammalia	Lagamorpha	Leporidae	*Sylvilagus audubonii*	Desert Cottontail	Syl_aud	Known	3,652,166	1,281,447	69.3
Mammalia	Lagamorpha	Leporidae	*Sylvilagus bachmani*	Brush Rabbit	Syl_bac	Suspected	416,353	16,458	0.9
Mammalia	Lagamorpha	Leporidae	*Sylvilagus floridanus*	Eastern Cottontail	Syl_flo	Suspected	5,968,739	1,278,473	73.0
Mammalia	Lagamorpha	Leporidae	*Sylvilagus nutallii*	Mountain Cottontail	Syl_nut	Suspected	2,066,991	100,694	5.7
Mammalia	Lagamorpha	Leporidae	*Sylvilagus palustris*	Marsh Rabbit	Syl_pal	Suspected	359,134	157,113	9.0
Mammalia	Lagamorpha	Leporidae	*Sylvilagus robustus*	Robust Cottontail	Syl_rob	Suspected	38,044	23,585	1.3
Mammalia	Rodentia	Cricetidae	*Neotoma albigula*	White-throated Woodrat	Neo_alb	Suspected	834,344	343,234	19.6
Mammalia	Rodentia	Cricetidae	*Neotoma cinerea*	Bushy-tailed Woodrat	Neo_cin	Suspected	2,382,393	96,254	6.7
Mammalia	Rodentia	Cricetidae	*Neotoma devia*	Arizona Woodrat	Neo_dev	Suspected	73,684	51,347	2.9
Mammalia	Rodentia	Cricetidae	*Neotoma floridana*	Key Largo Woodrat	Neo_flo	Suspected	1,871,082	409,898	23.4
Mammalia	Rodentia	Cricetidae	*Neotoma lepida*	Desert Woodrat	Neo_lep	Suspected	984,416	225,515	12.9
Mammalia	Rodentia	Cricetidae	*Neotoma leucodon*	White-toothed Woodrat	Neo_leu	Suspected	1,076,919	532,389	28.3
Mammalia	Rodentia	Cricetidae	*Neotoma macrotis*	Large-eared Woodrat	Neo_mac	Suspected	160,400	22,151	2.1
Mammalia	Rodentia	Cricetidae	*Neotoma mexicana*	Mexican Woodrat	Neo_mex	Suspected	1,725,668	396,435	22.6
Mammalia	Rodentia	Cricetidae	*Neotoma microps*	Southern Plains Woodrat	Neo_mic	Known	1,063,792	855,226	48.8
Mammalia	Rodentia	Cricetidae	*Neotoma stephensi*	Stephen's Woodrat	Neo_ste	Suspected	226,739	87,415	5.0
Mammalia	Rodentia	Heteromyidae	*Dipodomys agilis*	Agile Kangaroo Rat	Dip_agi	Suspected	38,595	4,509	0.3
Mammalia	Rodentia	Heteromyidae	*Dipodomys compactus*	Gulf Coast Kangaroo Rat	Dip_com	Suspected	49,441	48,289	2.8
Mammalia	Rodentia	Heteromyidae	*Dipodomys deserti*	Dessert Kangaroo Rat	Dip_des	Suspected	307,683	163,252	9.3
Mammalia	Rodentia	Heteromyidae	*Dipodomys elator*	Texas Kangaroo Rat	Dip_ela	Suspected	10,768	10,768	0.6
Mammalia	Rodentia	Heteromyidae	*Dipodomys merriami*	Merriam's Kangaroo Rat	Dip_mer	Suspected	1,423,668	678,724	38.7
Mammalia	Rodentia	Heteromyidae	*Dipodomys microps*	House Rock Valley Kangaroo Rat	Dip_mic	Suspected	392,306	81,458	4.6
Mammalia	Rodentia	Heteromyidae	*Dipodomys nelsoni*	Nelson's Kangaroo Rat	Dip_nel	Suspected	192,593	5,386	0.3
Mammalia	Rodentia	Heteromyidae	*Dipodomys ordii*	Ord's Kangaroo Rat	Dip_ord	Suspected	3,339,852	860,672	49.1
Mammalia	Rodentia	Heteromyidae	*Dipodomys panamintinus*	Panamint Kangaroo Rat	Dip_pan	Suspected	60,988	16,663	1.0
Mammalia	Rodentia	Heteromyidae	*Dipodomys spectabilis*	Banner-tailed Kangaroo Rat	Dip_spe	Suspected	523,875	292,001	16.7
Mammalia	Rodentia	Sciuridae	*Cynomys gunnisoni*	Gunnison's Prairie Dog	Cyn_gun	Suspected	309,504	77,244	4.4
Mammalia	Rodentia	Sciuridae	*Cynomys leucurus*	White-tailed Prairie Dog	Cyn_leu	Suspected	269,599	8,934	0.5
Mammalia	Rodentia	Sciuridae	*Cynomys ludovicianus*	Arizona Black-tailed Prairie Dog	Cyn_lud	Known	1,817,415	790,199	45.1
Mammalia	Rodentia	Sciuridae	*Spermophilus beecheyi*	California Ground Squirrel	Spe_bee	Known	462,986	25,396	1.4
Mammalia	Rodentia	Sciuridae	*Spermophilus mexicanus*	Mexican Ground Squirrel	Spe_mex	Suspected	715,741	503,163	28.7
Mammalia	Rodentia	Sciuridae	*Spermophilus mohavensis*	Mohave Ground Squirrel	Spe_moh	Suspected	20,552	17,869	1.0
Mammalia	Rodentia	Sciuridae	*Spermophilus spilosoma*	Spotted Ground Squirrel	Spe_spi	Suspected	1,712,203	824,260	47.0
Mammalia	Rodentia	Sciuridae	*Spermophilus tereticaudus*	Round-tailed Ground Squirrel	Spe_ter	Suspected	309,357	184,523	10.5
Mammalia	Rodentia	Sciuridae	*Spermophilus tridecemlineatus*	Thirteen-lined Ground Squirrel	Spe_tri	Suspected	3,679,468	605,633	4.6
Mammalia	Rodentia	Sciuridae	*Spermophilus variegatus*	Rock Squirrel	Spe_var	Suspected	2,304,502	809,092	46.2
Reptilia	Squamata	Colubridae	*Drymarchon couperi*	Eastern Indigo Snake	Dry_cou	Suspected	432,391	289,735	16.5
Reptilia	Squamata	Colubridae	*Drymarchon melanurus*	Central America Indigo Snake	Dry_mel	Suspected	667,459	123,957	7.1
Reptilia	Squamata	Viperidae	*Crotalus admanteus*	Eastern Diamond-back Rattlesnake	Cro_adm	Suspected	305,846	153,769	8.8
Reptilia	Squamata	Viperidae	*Crotalus atrox*	Western Diamond-back Rattlesnake	Cro_atr	Known	1,775,279	1,078,095	61.5
Reptilia	Squamata	Viperidae	*Crotalus cerastes*	Sidewinder	Cro_cer	Suspected	320,351	191,640	10.9
Reptilia	Squamata	Viperidae	*Crotalus lepidus*	Rock Rattlesnake	Cro_lep	Suspected	803,968	299,736	17.1
Reptilia	Squamata	Viperidae	*Crotalus mitchellii*	Speckled Rattlesnake	Cro_mit	Suspected	378,170	183,401	10.5
Reptilia	Squamata	Viperidae	*Crotalus molossus*	Black-tailed Rattlesnake	Cro_mol	Suspected	1,448,994	501,637	28.6
Reptilia	Squamata	Viperidae	*Crotalus scutulatus*	Mojave Rattlesnake	Cro_scu	Suspected	935,404	404,583	23.1
Reptilia	Squamata	Viperidae	*Crotalus viridis*	Western Rattlesnake	Cro_vir	Suspected	1,800,253	690,974	39.4
Reptilia	Squamata	Viperidae	*Sistrurus catenatus*	Massasauga	Sis_cat	Suspected	1,230,385	570,117	32.5
Reptilia	Squamata	Viperidae	*Sistrurus milaris*	Pigmy Rattlesnake	Sis_mil	Suspected	1,072,665	219,927	12.6
Reptilia	Testudines	Emydidae	*Terrapene ornata*	Ornate Box Turtle	Ter_orn	Known	2,241,202	1,093,432	62.4
Reptilia	Testudines	Testudinidae	*Gopherus agassizii*	Desert Tortoise	Gop_aga	Known	369,595	206,721	11.8
Reptilia	Testudines	Testudinidae	*Gopherus polyphemus*	Gopher Tortoise	Gop_pol	Known	271,398	125,148	7.1
