# Supplementary material for: Assessment of the Geographic Distribution of Ornithodoros turicata (Argasidae): Climate Variation and Host Diversity
Source: PLoS Negl Trop Dis. 2016 Feb 1;10(2):e0004383. doi: 10.1371/journal.pntd.0004383 (PMC4734830; doi:10.1371/journal.pntd.0004383)
Supplement: S4 Table — (PDF) [file pntd.0004383.s009.pdf]

S4 Table. Literature search of tick-borne relapsing fever spirochetes as an indicator of *O. turicata* distribution

Detection method	State	County	Location	Year	Isolate	Reference
Infected *O. turicata*	TX	Gaines	-	1950	-	[1]
Infected *O. turicata*	TX	Cochran	-	1950	-	[1]
Infected *O. turicata*	TX	Dawson	-	1950	-	[1]
Infected *O. turicata*	TX	Terry	-	1950	-	[1]
Infected *O. turicata*	TX	Martin	-	1950	-	[1]
Infected *O. turicata*	TX	Winkler	-	1950	-	[1]
Infected *O. turicata*	TX	Ward	-	1950	-	[1]
Infected *O. turicata*	TX	Val Verde	-	1950	-	[1]
Infected *O. turicata*	TX	Edwards	-	1950	-	[1]
Infected *O. turicata*	TX	Maverick	-	1950	-	[1]
Infected *O. turicata*	TX	Zavala	-	1950	-	[1]
Infected *O. turicata*	TX	Dimmit	-	1950	-	[1]
Infected *O. turicata*	TX	Uvalde	-	1950	-	[1]
Infected *O. turicata*	TX	Medina	-	1950	-	[1]
Infected *O. turicata*	TX	Bexar	-	1950	-	[1]
Infected *O. turicata*	TX	Hays	-	1950	-	[1]
Infected *O. turicata*	TX	Travis	-	1950	-	[1]
Infected *O. turicata*	TX	Williamson	-	1950	-	[1]
Infected *O. turicata*	TX	Burnet	-	1950	-	[1]
Infected *O. turicata*	TX	Bell	-	1950	-	[1]
Infected *O. turicata*	TX	Coryell	-	1950	-	[1]
Infected *O. turicata*	TX	Lampasas	-	1950	-	[1]
Infected *O. turicata*	TX	San Saba	-	1950	-	[1]
Infected *O. turicata*	TX	Brown	-	1950	-	[1]
Infected *O. turicata*	TX	Comanche	-	1950	-	[1]
Infected *O. turicata*	TX	Lano	-	1950	-	[1]
Infected *O. turicata*	TX	Gillespie	-	1950	-	[1]
Infected *O. turicata*	TX	Kimble	-	1950	-	[1]
Infected *O. turicata*	TX	Menard	-	1950	-	[1]
Infected *O. turicata*	TX	Tom Green	-	1950	-	[1]
Infected *O. turicata*	TX	Sutton	-	1950	-	[1]
Infected *O. turicata*	TX	Shackelford	-	1950	-	[1]
Infected *O. turicata*	TX	Dallas	-	1950	-	[1]
Infected *O. turicata*	TX	Tarrant	-	1950	-	[1]
Infected *O. turicata*	TX	Denton	-	1950	-	[1]
Retrospective diagnosis of *B. turicatae* infection	TX	Mills	"Northwest corner of Mills county"	1950, 1938	-	[1,2]

Retrospective diagnosis of *B. turicatae* infection	TX	Kimble	"12 miles west of Junction"	1938	-	[2]
Retrospective diagnosis of *B. turicatae* infection	TX	Crockett	-	1995	+	[3]
Domestic canine	TX	Taylor	-	1995	-	[3]
Retrospective diagnosis of *B. turicatae* infection	TX	Starr	"Along the Rio Grande"	1995	-	[3]
Infected *O. turicata*	TX	Atascosa	-	2005	+	[4]
B. turicatae isolated from domestic canine	TX	Wichita	Wichita Falls	2007	-	[5]
B. turicatae isolated from domestic canine	TX	Clay	Henrietta	2005, 2007	+	[4,5]
B. turicatae isolated from domestic canine	TX	Lubbock	Slaton	2005, 2007	+	[4,5]
Infected *O. turicata*	TX	Real	-	2005	+	[4]
Infected *O. turicata*	KS	Clark	Ashland	1943	-	[7]
B. turicatae isolated from domestic canine	FL	Sumner	"cypress swamp"	2005, 1994	+	[4,8]

References

1. Eads RB, Henderson HE, McGregor T, Irons JV (1950) Relapsing fever in Texas; distribution of laboratory confirmed cases and the arthropod reservoirs. Am J Trop Med Hyg 30: 73-76.
2. Francis E (1938) Longevity of the tick *Ornithodoros turicata* and of *Spirochaeta recurrentis* with this tick. Publ Hlth Rep 53: 2220-2241.
3. Rawlings JA (1995) An overview of tick-borne relapsing fever with emphasis on outbreaks in Texas. Tex Med 91: 56-59.
4. Schwan TG, Raffel SJ, Schrumpf ME, Policastro PF, Rawlings JA, et al. (2005) Phylogenetic analysis of the spirochetes *Borrelia parkeri* and *Borrelia turicatae* and the potential for tick-borne relapsing fever in Florida. J Clin Microbiol 43: 3851-3859.
5. Whitney MS, Schwan TG, Sultemeier KB, McDonald PS, Brillhart MN (2007) Spirochetemia caused by *Borrelia turicatae* infection in 3 dogs in Texas. Vet Clin Pathol 36: 212-216.
6. Davis H, Vincent JM, Lynch J (2002) Tick-borne relapsing fever caused by *Borrelia turicatae*. Pediatr Infect Dis J 21: 703-705.
7. Davis GE (1943) Relapsing fever: the tick *Ornithodoros turicata* as a spirochetal reservoir. Pub Health Rep 58: 839-842.
8. Breitschwerdt EB, Nicholson WL, Kiehl AR, Steers C, Meuten DJ, et al. (1994) Natural infections with *Borrelia* spirochetes in two dogs from Florida. J Clin Microbiol 32: 352-357.
